# Functionalization of Nanomaterials for Energy Storage and Hydrogen Production Applications

**DOI:** 10.3390/ma18040768

**Published:** 2025-02-10

**Authors:** Mohamed Salaheldeen, Ahmed M. Abu-Dief, Tarek El-Dabea

**Affiliations:** 1 Department of Polymers and Advanced Materials, Faculty of Chemistry, University of the Basque Country, UPV/EHU, 20018 San Sebastian, Spain; 2 Department of Applied Physics I, EIG, University of the Basque Country, UPV/EHU, 20018 San Sebastian, Spain; 3 Physics Department, Faculty of Science, Sohag University, Sohag 82524, Egypt; 4 Chemistry Department, College of Science, Taibah University, P.O. Box 344, Madinah 42353, Saudi Arabia; 5 Department of Chemistry, Faculty of Science, Sohag University, Sohag 82524, Egypt; 6 Chemistry Department, Faculty of Science, King Salman International University, Ras Sudr, Sinai 46612, Egypt; tarekeldabea@gmail.com

**Keywords:** functionalization, nanomaterials, energy storage, hydrogen production, supercapacitors

## Abstract

This review article provides a comprehensive overview of the pivotal role that nanomaterials, particularly graphene and its derivatives, play in advancing hydrogen energy technologies, with a focus on storage, production, and transport. As the quest for sustainable energy solutions intensifies, the use of nanoscale materials to store hydrogen in solid form emerges as a promising strategy toward mitigate challenges related to traditional storage methods. We begin by summarizing standard methods for producing modified graphene derivatives at the nanoscale and their impact on structural characteristics and properties. The article highlights recent advancements in hydrogen storage capacities achieved through innovative nanocomposite architectures, for example, multi-level porous graphene structures containing embedded nickel particles at nanoscale dimensions. The discussion covers the distinctive characteristics of these nanomaterials, particularly their expansive surface area and the hydrogen spillover effect, which enhance their effectiveness in energy storage applications, including supercapacitors and batteries. In addition to storage capabilities, this review explores the role of nanomaterials as efficient catalysts in the hydrogen evolution reaction (HER), emphasizing the potential of metal oxides and other composites to boost hydrogen production. The integration of nanomaterials in hydrogen transport systems is also examined, showcasing innovations that enhance safety and efficiency. As we move toward a hydrogen economy, the review underscores the urgent need for continued research aimed at optimizing existing materials and developing novel nanostructured systems. Addressing the primary challenges and potential future directions, this article aims to serve as a roadmap to enable scientists and industry experts to maximize the capabilities of nanomaterials for transforming hydrogen-based energy systems, thus contributing significantly to global sustainability efforts.

## 1. Introduction

**Scheme 1:**
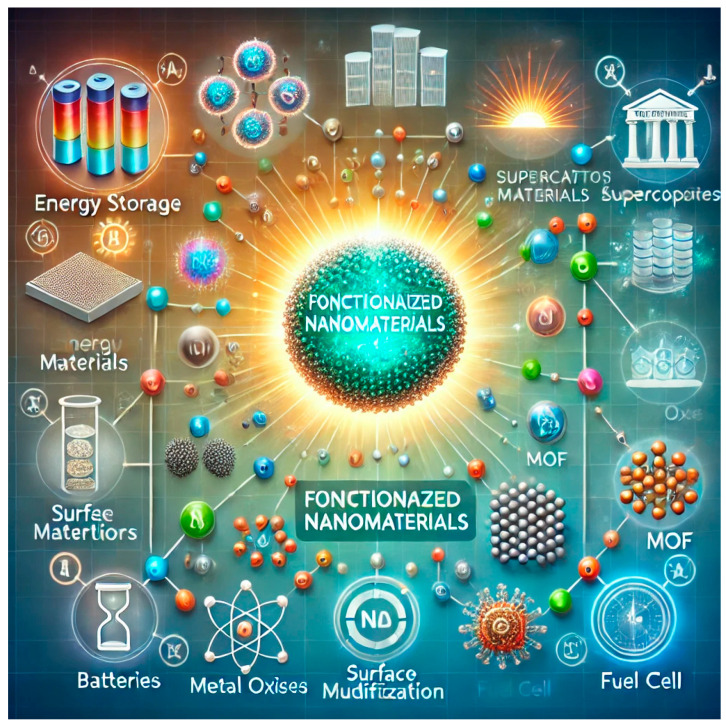
Diagrammatic scheme showing functionalization of nanomaterials and their different energy applications.

Today’s worldwide energy sector confronts extraordinary hurdles, mainly due to diminishing fossil fuel resources and the urgent need to address climate change impacts [1,2]. This scenario has precipitated a paradigm shift towards sustainable and clean energy sources, with hydrogen emerging as a potential key player in achieving a carbon-neutral energy future [3]. Being the most common element found in the universe, hydrogen shows promising characteristics for energy transport, featuring exceptional energy content by weight (142 MJ/kg), zero-emission combustion, multiple production methods, and capacity for substantial energy storage [4]. However, despite these benefits, widespread implementation of hydrogen-based technologies encounters major obstacles, especially regarding its generation and containment [5,6]. Current methods for producing hydrogen, such as steam methane reforming (SMR), electrolysis, and photoelectrochemical water splitting, each present unique advantages and limitations [7,8]. The advancement of efficient, affordable, and environmentally friendly benign production methods that can be scaled to meet global energy demands remains a critical challenge [8]. Similarly, hydrogen storage presents a significant bottleneck in the hydrogen energy ecosystem. Current storage methods, such as liquefaction, compression, and solid-state storage, each come with inherent challenges related to energy efficiency, storage density, and practical application [9,10]. The enhancement of advanced materials as well as innovations for safe, high-density, and reversible hydrogen storage is a key research focus in the field [11,12,13]. Nanomaterials have emerged as potential game-changers in addressing these challenges owing to their distinctive characteristics, such as a high surface-to-volume ratio, adjustable electronic and optical characteristics, enhanced catalytic activity, and improved kinetics for hydrogen absorption and desorption [14]. These characteristics position nanomaterials as ideal candidates for developing more efficient catalysts, high-capacity storage constituents, as well as advanced energy storage systems [15]. Functionalization, the process for modifying nanomaterials with specific chemical groups or molecules, offers a powerful approach to further enhance their performance in energy applications [16]. By carefully engineering the surface chemistry as a framework for nanomaterials, researchers may potentially increase catalytic activity and selectivity for H_2_ production, enhance H_2_ storage (capacity and kinetics), improve the stability and cyclability for energy storage devices, and tailor electronic properties for specific applications [17]. This review article offers an in-depth summary of recent developments in the functionalization of nanomaterials for energy storage and hydrogen production. We examine various classes of nanomaterials, with a focus on metal oxides, carbon-based materials, as well as hybrid nanostructures, along with different functionalization strategies and their impact on material properties. The review encompasses applications in electrocatalytic and photocatalytic hydrogen production, advancements in hydrogen storage materials, and broader energy storage applications, including batteries and supercapacitors (Scheme 1). Looking ahead, the field of functionalized nanomaterials for energy applications holds immense promise. Future research directions may include the development of multifunctional nanomaterials, exploration of bio-inspired functionalization strategies, leveraging artificial intelligence and machine learning for the design and optimization of materials, developing scalable manufacturing processes for industrial use, and integrating functionalized nanomaterials into real-world applications [18,19,20]. By addressing these challenges and opportunities, functionalized nanomaterials could significantly contribute to achieving a sustainable, hydrogen-powered energy economy. The goal of this review is to offer an in-depth evaluation of the status of the field and to highlight potential areas for future research and development.

## 2. Nanostructured Materials

Nanostructured materials exhibit considerable potential for H_2_ storage products because of their distinctive characteristics, including surface adsorption, inter- and intragranular boundary interactions, and bulk absorption capabilities [21]. The nanoscale dimensions of these materials significantly influence both (thermodynamics and kinetics) H_2_ absorption and dissociation systems. This influence is manifested through enhanced diffusion rates and reduced diffusion path lengths [21]. Furthermore, nanostructured materials offer the unique advantage of allowing independent control over material tailoring parameters, a feature not readily available in their bulk counterparts [22]. This level of control facilitates the creation and engineering of efficient, compact hydrogen containment systems with enhanced storage capabilities. The increased surface area-to-volume ratio, as well as the occurrence of numerous active sites in nanostructured materials, contribute to their enhanced H_2_ storage capacity and improved sorption kinetics [22]. Consequently, these materials are increasingly being explored for the improvement of next-generation H_2_ storage solutions that link the stringent supplies for practical applications in clean energy technologies.

## 3. Fundamentals and Pathway of Hydrogen Storage in Nanomaterials

**Figure 1:**
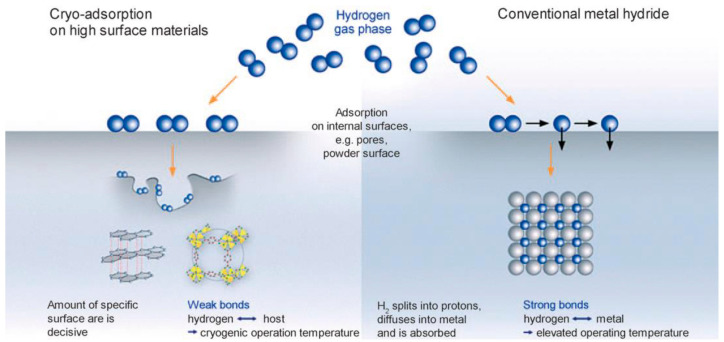
Graphic representations demonstrating the concepts for chemical and physical H_2_ storage [26].

Solid-state H_2_ storage in nanomaterials operates through two primary mechanisms: physical and chemical adsorption, utilizing specialized solid materials to capture and release hydrogen [23,24,25]. Physical adsorption materials, including MOFs and activated carbon, use their large surface areas and tiny pores to physically bind hydrogen molecules through van der Waals forces, performing optimally at low temperatures and being amenable to performance enhancement through structural modifications. In contrast, metal hydrides like TiFe alloys and LaNi_5_ form stable compounds through chemical reactions with hydrogen, absorbing heat during hydrogen uptake and releasing it during desorption, requiring precise temperature and pressure control for efficient operation. These complementary approaches offer different advantages for hydrogen storage, with physical adsorbents excelling in surface-based storage and metal hydrides providing chemical-bond-based storage solutions [26]. The fundamental mechanisms of adsorption are detailed in Figure 1 [26].

## 4. Optimizing Surface Properties for Improved Hydrogen Storage

Surface adjustment represents a crucial approach in optimizing nanomaterials’ hydrogen storage capabilities through three primary mechanisms: modifying surfaces through chemical functionalization, atomic doping, and engineered defect sites [27]. Chemical functionalization involves attaching specific functional groups (N-H, COOH groups, and metal nanoparticles) to nanomaterial surfaces, as demonstrated in carbon nanotubes (CNTs) modified with Pd and Pt particles, which achieved remarkable improvements in H_2_ storage capacity—up to 4.5 wt.% with Pd modification and 2.7 wt.% with Pt deposition through sc-CO_2_ treatment [28,29].

The significant increase in hydrogen adsorption capacity, about 4–5 times higher with respect to that of the pristine CNTs, was mainly attributed to the spillover mechanism. The presence of nano-sized Pt particles acting as catalysts for the dissociation of H_2_ into H atoms enhanced the diffusion process and promoted the subsequent chemisorption of hydrogen atom onto the active site on the CNT wall surface. However, as the decorated Pt was increased beyond 9.39 wt.%, descending hydrogen adsorption with increasing Pt loading was observed. With higher Pt loading, the surface of CNTs could be occupied by excess Pt particles, resulting in a decrease in the active sites for hydrogen adsorption. The beneficial effect of Pt decoration on the enhancement of hydrogen adsorption capacity via the hydrogen spillover mechanism has been reported elsewhere [30]. Hydrogen spillover involves the dissociation of molecular hydrogen (H_2_) into atomic hydrogen (H) on the surface of a Pt catalyst, followed by the migration of these hydrogen atoms onto an adjacent support material. This process consists of the following steps:

1. **Dissociation**: Pt, as a transition metal, has a high catalytic activity for H_2_ dissociation. It splits molecular hydrogen into atomic hydrogen.
2. **Migration**: The atomic hydrogen formed on the Pt surface migrates to the surface of the adjacent material.
3. **Adsorption**: The hydrogen atoms are adsorbed or stored on the surface or within the pores of the support material.

**Role of Pt Decoration**

1. **Catalytic Activity**: Pt nanoparticles or clusters provide active sites for the dissociation of H_2_, which is otherwise difficult for many materials to achieve directly.
2. **Spillover Enhancement**: Once atomic hydrogen forms on the Pt surface, it migrates to the support material (e.g., carbon, metal–organic frameworks (MOFs), or other porous materials). The decoration of Pt ensures efficient transfer of atomic hydrogen, enhancing the overall adsorption capacity.
3. **Increased Storage**: By enabling hydrogen to absorb high-surface-area materials via spillover, the total hydrogen storage capacity is significantly improved compared to the use of the support material alone.

**Beneficial Effects**

- **Higher Hydrogen Uptake**: The hydrogen storage capacity is greatly enhanced due to the synergistic effect of Pt and the support material.
- **Low-Pressure Adsorption**: The spillover mechanism facilitates hydrogen adsorption at lower pressures, which is advantageous for practical applications in hydrogen storage systems.
- **Material Versatility**: Pt decoration enables the use of materials that inherently have low or no catalytic activity for H_2_ dissociation.

**Figure 2:**
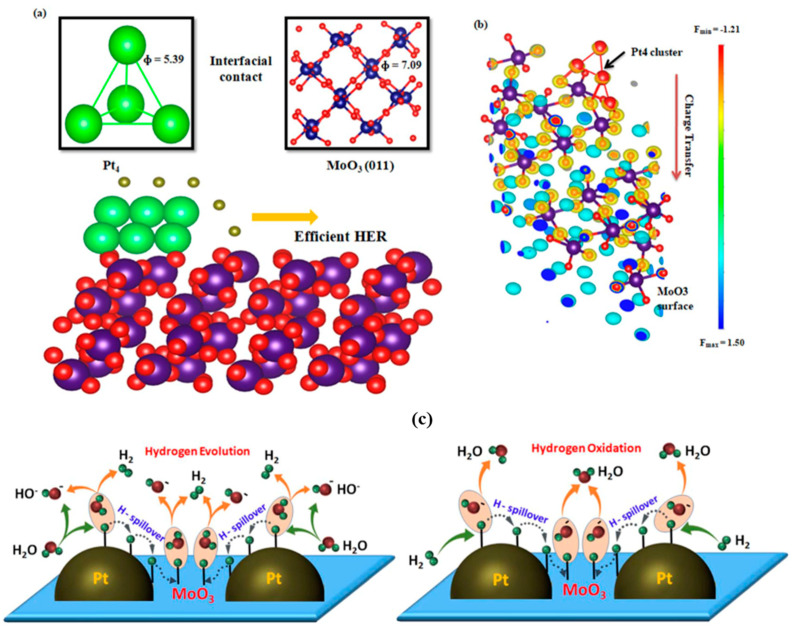
(**a**) Schematic illustration of the hydrogen spillover mechanism over a Pt_4_@MoO_3_ (011) surface. (**b**) Charge density difference plot across the Pt_4_@MoO_3_ (011) surface. (**c**) Schematic representation of the hydrogen spillover-induced HER/HOR mechanism in alkaline medium [31].

It is difficult to directly observe the electrocatalytic hydrogen spillover phenomenon. However, some theoretical concepts have recently been reported to prove the hydrogen spillover phenomenon. To explore additional theoretical insight enabling efficient hydrogen spillover for the hydrogen evolution reaction (HER), a work-function-dependent HER activity was performed to determine the kinetics of the interfacial spillover process on a Pt-supported β-MoO_3_ (011) surface. The work function of the Pt nanocluster was found to be F1 = 5.39 eV smaller than that of the β-MoO_3_ (011) surface work function (F2 = 7.09 eV), revealing charge transfer from Pt nanoclusters to the β-MoO_3_ surface, which could also be verified by a charge density difference plot, as shown in Figure 2a,b. The red colors show a charge loss region at the Pt cluster, whereas the blue color on the β-MoO_3_ (011) surface indicates a charge gain region, i.e., electrons are transferring from the Pt to the β-MoO_3_ (011) surface. Under alkaline conditions using KOH solution, the morphology and structure of the Pt_4_@ β-MoO_3_ (011) were stable. The difference in the free energy of Pt_4_@ β-MoO_3_ (011) under vacuum and alkaline media was 0.14 eV, which was lower and exhibited the feasibility of Pt_4_@ β-MoO_3_ (011) in alkaline media. The gradual decrease in HOR activity with increasing pH indicated that the reaction went through the hydrogen binding energy (HBE) mechanism. During the HER, H_2_O dissociates on metallic Pt and interfaces to form Pt–Had or (Pt/MoO_3_)–Had and OH−. Some of the adsorbed hydrogen migrates to the MoO_3_ sites due to hydrogen spillover, which thereby boosts the Volmer step. Finally, Had is formed at Pt and the interface, and MoO_3_ sites react with H_2_O to form H_2_ molecules, enhancing the Heyrovsky step, which leads to the high HER activity of Pt/MoO_3_-CNx-400. Similarly, for the HOR, H_2_ dissociates on Pt and interfaces to form Pt–Had and (Pt/MoO_3_)–H_ad_. Then, some adsorbed hydrogen may transfer to the MoO_3_ site to form MoO_3_–Had via the hydrogen spillover process. Had subsequently interacts with the OH− of the electrolyte to form H_2_O, which leads to the high HOR performance of the catalyst. A schematic representation of the hydrogen spillover-induced alkaline HER/HOR mechanism is shown in Figure 2c.

### Factors Contributing to the High Catalytic Performance of Pt/ MoO_3_-CNx-400

**Figure 3:**
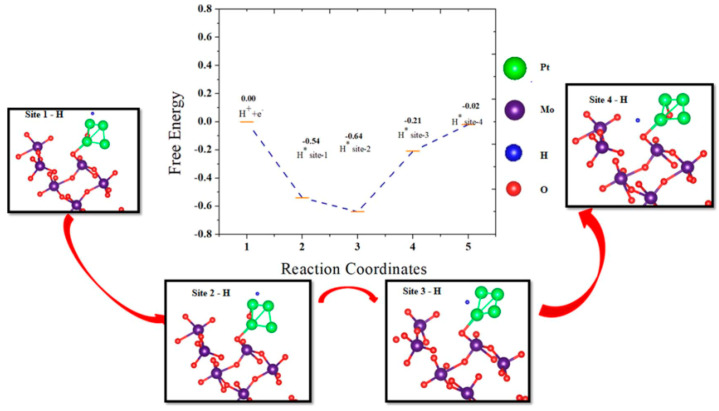
Schematic presentation of a free energy diagram for the HER on the surface of Pt@MoO_3_ (011). Optimized H* adsorption structures from site 1 to site 4 at different locations on Pt@MoO_3_ (011) are shown in the black boxes according to the red arrows [31].

**Figure 4:**
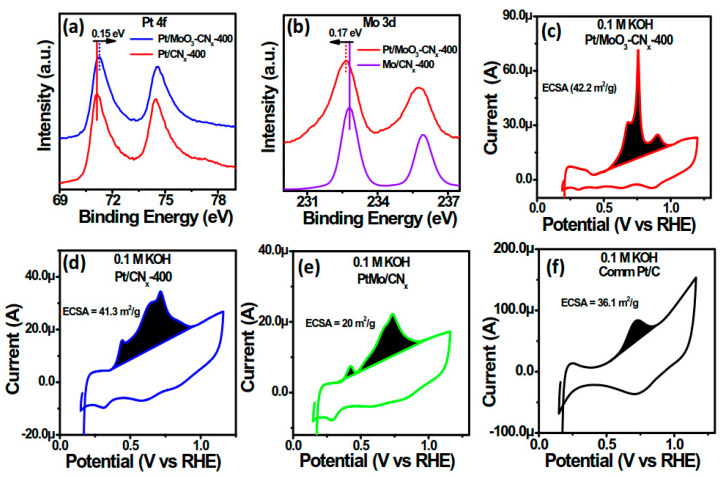
(**a**) Comparison of Pt 4f XPS spectra of Pt/MoO_3_-CNx-400 and Pt/CNx-400. (**b**) Comparison of Mo 3d XPS spectra of Pt/MoO_3_-CNx-400 and Mo/CNx-400. CO-stripping experiment of (**c**) Pt/MoO_3_-CNx-400, (**d**) Pt/CNx-400, (**e**) PtMo/CNx, and (**f**) comm Pt/C in base, respectively.

Several factors are responsible for the excellent HOR/HER activity of Pt/MoO_3_-CNx-400:

1. The hydrogen spillover is the most important factor for the high catalytic activity of Pt/MoO_3_-CNx-400. It was reported by Wang and co-workers that the smaller work function of nanoparticles compared to the surface reveals charge transfer from nanoparticles to the surface, which facilitates the hydrogen transfer process and subsequently boosts the HER [32]. It was found that the work function of the Pt nanocluster is 5.39 eV, which is smaller than the β-MoO_3_ (011) surface work function (F2 = 7.09 eV), revealing charge transfer from Pt nanoclusters to the b-MoO_3_ surface (Figure 2a). The charge density difference plot also suggests the same (Figure 2b). The energy problem diagram suggests that hydrogen spillovers occur from the Pt to the MoO_3_ sites, as shown in Figure 3, which illustrates that there is slightly higher adsorption energy at the Pt and interface sites, while MoO_3_ possesses weak adsorption energy. Therefore, hydrogen can easily adsorb at the Pt and interface sites and desorb from the MoO_3_ site. With less of an energy barrier, hydrogen can move from Pt or the interface to the MoO_3_ site instead of direct desorption occurring from Pt or interfaces, which thereby increases the HER/HOR activity.
2. The synergistic interaction among Pt, MoO_3_, and CNx components could play an important role in the high catalytic performance. The enhanced HER activity of the (Mo_3_S_13_)^2−^ cluster co-catalyst and WSe_2_ photocathode was due to the synergistic interaction between the components. The presence of synergistic interaction and electronic modulation was confirmed by the Pt 4f and Mo 3d XPS peak shifting of Pt/MoO_3_-CNx-400 compared to Pt/CNx-400 and Mo/CNx-400, respectively (Figure 4a,b). The figure shows a positive shift of Pt 4f of Pt/MoO_3_-CNx-400 compared to Pt/CNx-400 and a negative shift of Mo 3d of Pt/MoO_3_-CNx-400 compared to Mo/CNx-400, suggesting electron transfer from Pt to the MoO_3_ site. This indicates electronic modulation of Pt andMoO_3_ in the Pt/MoO_3_-CNx-400 composite.

1. The existence of interfaces as active catalytic sites has been reported by a number of groups. For example, Sun and co-workers reported that the interface between nickel and nickel nitrides boosts the HER/HOR activity of the catalyst [33]
2. Another important factor for the high activity of the catalyst is the ECSA, which is generally proportional to the electrochemical activity of a catalyst. CO-stripping experiments were performed to calculate the ECSAs for the catalysts (Figure 4c–f), and they showed that the highest ECSA value of 42.2 m^2^ g^−1^ was obtained for Pt/MoO_3_-CNx-400, as compared to other catalysts. The greater HER/HOR performance of Pt/MoO_3_-CNx-400 may have been due to the high ECSA of the catalyst. The integration of amine groups into metal–organic frameworks (MOFs) enhanced storage capacity through additional adsorption sites and stronger hydrogen bonding. Heteroatoms doping with elements like nitrogen, boron, and sulfur create polarized surface sites that strengthen H_2_ binding by electrostatic interactions, particularly evident in graphene-based materials, which exhibit exceptional surface area (2630 m^2^/g) as well as thermal stability (melting point 4510 K) [34]. The controlled introduction of surface defects and vacancies, exemplified by TiO_2_ nanotubes capable of forming host–guest compounds (TiO_2−x_H_2_) and defect-engineered boron nitride nanotubes (BNNTs), provides additional hydrogen-trapping sites and enhances surface reactivity [35,36]. This comprehensive surface engineering approach, combining multiple modification strategies, has demonstrated significant improvements in H_2_ storage capacity, binding energy, and system kinetics, though optimization of these techniques continues for practical applications.

## 5. Metal-Oxide Catalysts for (HER = Hydrogen Evolution Reaction): Mechanisms, Strategies, and Performance Optimization

**Figure 5:**
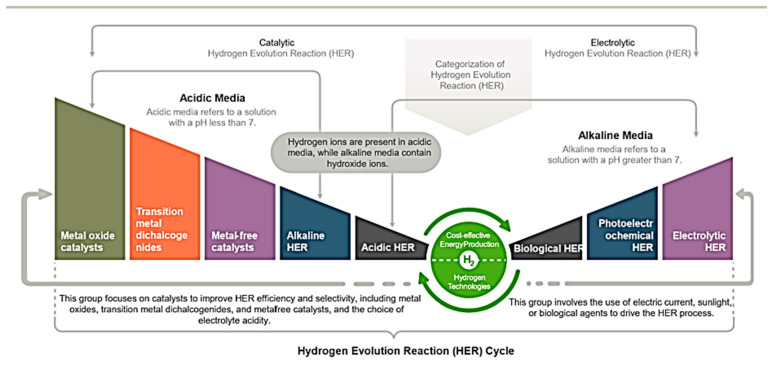
Classification of the hydrogen evolution reaction (HER) with permission from [46].

HER is a critical method in fresh energy technologies, involving two primary approaches: electrolytic and catalytic methods. In electrolytic HER, electricity drives water splitting, while catalytic HER employs various catalysts like metal oxides, alloys, and functional materials. The reaction occurs through electron transfer mechanisms (Volmer–Heyrovsky or Volmer–Tafel) with slight variations in acidic versus alkaline environments. In acidic media, the reaction proceeds more straightforwardly, whereas alkaline conditions require additional water bond dissociation, making the process kinetically more complex [37,38,39,40,41,42,43,44,45]. HER presents significant environmental and energy advantages. As a clean energy source, it generates only water vapor, offering a promising solution for reducing carbon emissions and mitigating climate change. Its high energy density makes it particularly attractive for transportation fuel applications, providing a potentially sustainable alternative to conventional fossil fuel technologies, as shown in Figure 5 [46]. HER encompasses multiple methodological approaches, including electrolytic, photoelectrochemical, and biological processes (Table 1). This section examines HER methods and their applications in electrocatalysis and renewable energy. Various catalysts enhance HER performance: ruthenium and molybdenum oxides show strong catalytic activity, while molybdenum and tungsten disulfides present promising results. Novel carbon-based catalysts like nitrogen-doped carbon nanotubes and graphene offer metal-free alternatives. The process works in both alkaline and acidic solutions, with alkaline conditions reducing costs and improving stability, while acidic environments boost activity and selectivity [44]. Metal oxide catalyst performance varies significantly. Copper oxide achieves 135 mV overpotential, while molybdenum oxide reaches 150 mV. Nickel oxide leads in current density at 12.5 mA cm^−2^. Zinc oxide shows the highest durability at 105 h, and cobalt oxide demonstrates optimal reaction kinetics with a 54 mV dec^−1^ Tafel slope. A performance comparison of various metal oxides in the hydrogen evolution reaction (HER) is presented in Table 2. Research in materials science continues to focus on optimizing HER electrocatalysts’ activity, selectivity, and stability. By combining strategic approaches and refining catalyst design, researchers aim to advance hydrogen-based clean energy technologies, addressing global energy challenges and promoting sustainable power solutions [45,46].

## 6. Nanomaterials Used in Hydrogen Production

**Figure 6:**
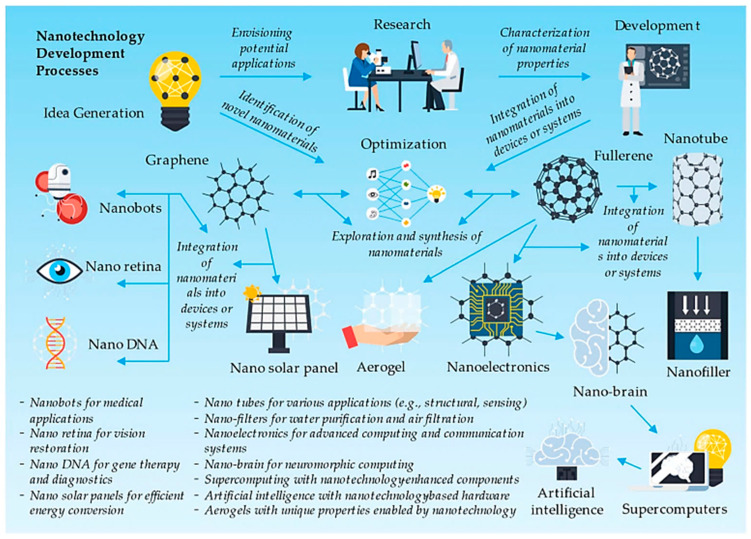
A systematic process for nanotechnology development, beginning with concept (creation and research) and growth, encompassing diverse efforts, as well as concluding with product commercialization and ongoing refinement with permission from [46].

Hydrogen can be produced through four distinct techniques: photoelectrochemical H_2_O splitting, solid material storage, photocatalytic hydrogen generation, and PEM fuel cells. The various methods for hydrogen generation, covering electrochemical, photoelectrochemical, photocatalytic, and electro-photochemical approaches are represented in Table 3. In photocatalysis, hydrogen and oxygen are produced when light creates electrons and holes in the semiconductor’s energy bands, triggering a redox reaction. For photocatalysts to work effectively, they need three key characteristics: they must have appropriate band gaps and structures that can harness sunlight or UV radiation to derive the half-reactions for hydrogen and oxygen; they should efficiently transport electrons and holes while minimizing their recombination; and they need to provide an extensive surface area to maximize their catalytic performance. The field of photoelectrochemical (PEC) water splitting began with Fujishima and Honda’s breakthrough using a TiO_2_ anode and Pt cathode system [47]. Later, Bard advanced this work by developing a photocatalytic system using semiconductor particles and powders [48]. TiO_2_ emerged as the leading semiconductor for PEC water splitting, though its wide band gap of 3.2 eV limited its ability to use visible and infrared light. To address this limitation, researchers developed (metal and non-metal) doping techniques to narrow the band gap as well as enable vis- light absorption [49,50]. Significant advances came with C-doped TiO_2_ nanocrystalline films, which achieved impressive conversion rates and worked under visible light [51]. Other important developments included combining TiO_2_ with fluorine-doped tin dioxide and exploring ZnO as an alternative semiconductor [52,53]. Researchers have investigated numerous nanomaterials for photocatalytic hydrogen production, including CdS; SiC; CuInSe_2_; and various metal oxides like Nb_2_O_5_, Ta_2_O_5_, and WO_3_ [54,55]. To overcome band gap limitations that reduced hydrogen production [56], scientists explored various enhancement techniques, including noble metal doping (primarily with Pt, though less expensive alternatives like Ag and Ru were also studied) and the use of co-catalysts [57]. Co-catalysts have proven particularly effective in improving water splitting efficiency. Various materials have been employed as reduction co-catalysts, including transition metals, metal oxides, and noble metals, which work by capturing electrons [58]. For oxidation, materials like IrO_2_ and RuO_2_ have been effective as hole-trapping co-catalysts. Some researchers have even developed dual co-catalyst systems, such as Pt-Ag_2_S and Pt-CuS combinations; these have demonstrated potential in enhancing the separation of photogenerated charges and boosting hydrogen production [59]. The development process includes preclinical and clinical assessments, regulatory approvals, commercialization, post-market surveillance, and ongoing advancements in nanotechnology-based products and applications (Figure 6).

## 7. Advanced Insights into Photocatalytic Hydrogen Evolution Reaction

Hydrogen stands out as an extremely promising replacement for non-renewable energy resources because of its remarkable energy density as well as vast availability. However, the conventional thermal processes for hydrogen production are often energy-intensive and offset its environmental benefits [60,61].

In recent years, alternative methods such as thermochemical cycles, solar-driven water splitting, electrolytic processes, and bio-hydrogen production have gained traction. Among these, photocatalytic water splitting has become a promising method for a particularly attractive strategy due to its sustainability and ability to directly transform solar energy into chemical energy. Photocatalytic H_2_ growth is a sophisticated process governed by a sequence of interconnected photophysical and photochemical mechanisms:

1. Photon Absorption: Incident photons, through energy equal to or higher than the photocatalyst’s band hole, excite electrons, generating electron–hole pairs.
2. Photoexcitation: Electrons are elevated from the valence band (VB) to the conduction band (CB), creating vacancies or holes in the VB, which generates an energy disparity essential for driving redox reactions.
3. Charge Carrier Dynamics: Efficient separation of charge carriers and transport are critical to minimizing electron–hole recombination, which competes with the catalytic reaction pathway.
4. Catalytic Redox Reactions: Electrons in the conduction band (CB) reduce the protons (H^+^) adsorbed on the catalyst surface to generate molecular hydrogen (H_2_), while the holes in the valence band (VB) oxidize water molecules, leading to the formation of oxygen [62].

**Figure 7:**
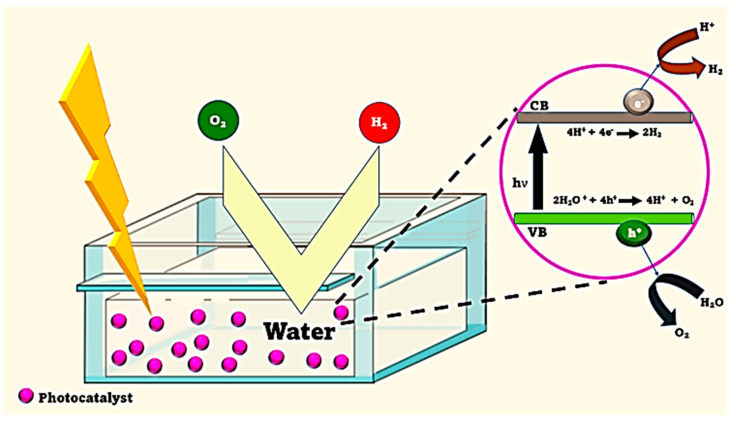
The process of photocatalytic water splitting for hydrogen production involves the use of light energy to drive the decomposition of water into hydrogen and oxygen gases, with permission from [67].

Despite the promise of this technology, its practical efficiency is hindered by several intrinsic and extrinsic factors. A major limitation lies in the narrow light absorption range of many conventional photocatalysts, primarily due to their large band gaps, which restrict utilization of the visible light spectrum, the dominant component of solar radiation. Additionally, the recombination of photogenerated electron–hole pairs significantly diminishes the availability of active carriers for catalytic reactions, reducing overall performance [63]. Research has identified and explored a variety of photocatalysts, including TiO_2_, WO_3_, MoS_2_, CdS, g-C_3_N_4_, Cu_2_O, ZnIn_2_S_4_, and CdIn_2_S_4_. Each material presents a trade-off between band gap properties and catalytic efficiency. Wide-band-gap semiconductors, while stable and durable, demonstrate limited absorption of visible light, restricting their efficiency in solar-to-hydrogen conversion. Conversely, narrow-band-gap semiconductors efficiently harness visible light but often lack sufficient oxidation power, limiting their applicability in comprehensive water-splitting systems. These challenges necessitate advanced strategies to enhance both light-harvesting efficiency and catalytic activity. Recent breakthroughs in photocatalyst design have focused on novel techniques to overcome these barriers. LSPR (localized surface plasmon resonance) has been utilized to improve light trapping and extend the absorption spectrum, significantly boosting photocatalytic performance [64]. Other strategies, such as elemental doping, heterojunction construction, and co-catalyst integration, have further refined the charge separation and catalytic efficiency of photocatalysts. For example, coupling g-C_3_N_4_ with platinum oxide (PtO) has demonstrated enhanced hydrogen evolution rates and superior photostability by facilitating efficient charge transfer and maximizing active site availability [65]. A noteworthy shift in the field involves transitioning from precious metals to earth-abundant materials like transition metal phosphides (TMPs). These compounds not only offer cost-effectiveness and sustainability, but also serve dual roles as co-catalysts and active photocatalytic sites. TMPs demonstrate remarkable proton reduction capabilities, contributing to higher hydrogen yields while maintaining scalability and economic feasibility [66]. In summary, photocatalytic hydrogen evolution represents a cutting-edge solution at the intersection of material science, quantum mechanics, and renewable energy technologies. Advancing this field requires continued exploration of novel photocatalyst architectures, dynamic charge management strategies, and scalable synthesis methods to bridge the gap between laboratory efficiency and real-world application. Through such innovations, photocatalysis could play a transformative role in achieving global clean energy goals. Figure 7 illustrates the process of the photocatalytic splitting of water for hydrogen production [67]. Table 4 presents the use of MOFs in photocatalytic H_2_ generation [68], and different metal free catalysts are described in the last section of Table 5, along with its H_2_ productivity rate. It also summarizes the different photocatalytic HERs based on non-noble-metal catalysts.

## 8. The Use of Magnetic Nanomaterials for Hydrogen Storage

**Figure 8:**
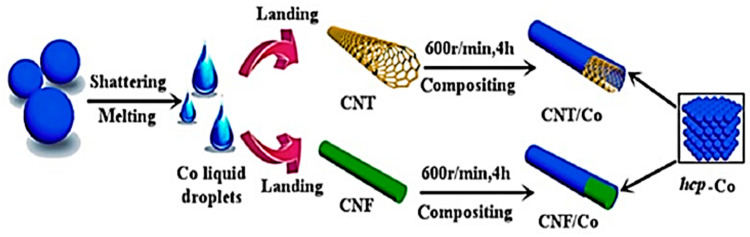
The process of creating magnetic core–shell structures of CNT/Co and CNF/Co is carried out using a ball-milling technique, with permission from [84].

Hydrogen presents a promising renewable energy solution, offering a clean alternative that can help combat global warming and alleviate the depletion of fossil fuel reserves [70]. Hydrogen has several advantages, such as being environmentally friendly, safe, versatile, and having a high energy density [71]. Hydrogen can be utilized in fuel cells, where its chemical energy is directly transformed into electricity, water, and heat, positioning it as a compelling alternative to fossil fuels [72]. Hydrogen storage is a critical aspect of the hydrogen economy, and various methods are being investigated, including metal hydrides [73], complex and chemical hydrides [74], adsorbents [75], nanomaterials [76], polymer nanocomposites, and metal–organic structures [77]. The magnetic properties of materials play a crucial role in determining their hydrogen storage capabilities through various fundamental mechanisms. In ferromagnetic materials, the spin-polarized electronic states create distinct absorption sites for hydrogen atoms, leading to modified storage characteristics compared to their non-magnetic counterparts. The presence of magnetic exchange interactions significantly alters the local electronic environment around potential hydrogen binding sites, which directly affects the material’s ability to absorb and retain hydrogen molecules. Recent studies on transition metal-based systems, particularly those containing Fe, Ni, and Co, have demonstrated enhanced storage capacities of up to 30% when in their ferromagnetic state. This enhancement is attributed to several factors: the spin-polarized electronic structure creates modified binding energies for hydrogen, the magnetic ordering can lead to favorable local lattice distortions, and the altered electronic structure facilitates improved electron transfer capabilities. Additionally, magnetic nanomaterials present unique opportunities for hydrogen storage due to their high surface-to-volume ratios and tunable magnetic properties. The correlation between magnetic properties and hydrogen storage is particularly evident in the modified activation barriers for hydrogen dissociation and the creation of enhanced diffusion pathways within the material’s structure. Understanding these magnetic-hydrogen storage relationships is crucial for the rational design of new storage materials and the optimization of existing systems for clean energy applications [78,79]. As hydrogen does not naturally exist in its pure form, it is produced through various methods, including steam reforming, electrolysis, thermochemical cycles, photobiological water splitting [79], photocatalytic water splitting [80], fermentative hydrogen production, and enzymatic hydrogen generation. Nanomaterials, particularly core–shell magnetic nanomaterials, have recently gained attention for hydrogen storage applications due to their distinct characteristics like surface adsorption, inter-grain boundaries, and bulk absorption [81,82]. These nanomaterials can regulate the adsorption and decomposition of hydrogen by affecting the diffusion rate [83]. Studies have shown that carbon-based nanoparticles with core–shell structures, such as CNF/Co and CNT/Co, can exhibit superior hydrogen storage capacities under mild conditions, as displayed in Figure 8 [84]. The creation of efficient and economical hydrogen storage solutions is vital for the widespread use of hydrogen as a clean energy carrier, and ongoing research in this field is focused on investigating novel nanomaterial-based methods.

## 9. Metal Oxide Nanomaterials for Sustainable Energy Applications

### 9.1. Energy Storage

Energy storage is crucial in modern times for balancing electricity supply and demand, as well as maintaining grid stability and reliability [85]. It allows for the integration of renewable energy sources like solar and wind energy by storing excess energy during periods of high production and discharging it when production is low or demand is high. These systems also improve power quality management, regulate frequency, and perform peak shaving, enhancing the efficiency and robustness of the electrical grid. Additionally, they support the adoption of electric vehicles by providing the required charging infrastructure and increasing driving ranges [86]. Nanomaterials play a pivotal role in advancing hydrogen production and storage technologies, offering innovative solutions to energy storage challenges.

**Hydrogen Production:**

Nanomaterials, particularly graphene-based catalysts, have been extensively researched for their efficiency in water splitting—a primary method of hydrogen generation. Graphene’s unique properties enhance the catalytic process, leading to more efficient hydrogen production [87].

**Hydrogen Storage:**

In terms of storage, nanostructured materials such as metal–organic frameworks (MOFs) and carbon nanotubes (CNTs) have shown significant promise. MOFs, with their high surface areas and tunable structures, can adsorb substantial amounts of hydrogen, making them suitable for practical applications. While MOFs exhibit great potential, challenges such as low hydrogen uptake and slow-release rates must be addressed in order for them to become practical hydrogen storage materials. Ongoing research aims to overcome these hurdles and unlock the full potential of MOFs in advancing hydrogen energy technologies. Similarly, CNTs have been explored for hydrogen storage due to their high surface area and unique structural properties, which facilitate hydrogen adsorption. CNTs have been studied both as catalysts for hydrogen production and as components of hydrogen storage materials. Researchers have developed CNT-based catalysts for processes like steam methane reforming, a common method of hydrogen production. Additionally, CNTs have been incorporated into hydrogen storage materials, such as metal hydrides, to enhance their hydrogen uptake and release kinetics [88,89]. The unique properties of CNTs contribute to their appeal, including high strength, excellent electrical and thermal conductivity, and the ability to be functionalized for specific applications. However, challenges exist, and ongoing research delves into addressing these challenges while harnessing the advantages of CNTs for advancements in hydrogen energy. These advancements in nanomaterials not only improve the efficiency of hydrogen production and storage, but also contribute to the development of sustainable energy storage solutions. The distinctive properties of metallic nanoparticles make them pivotal in hydrogen-related applications. Their high surface area and reactivity are harnessed for catalyzing reactions essential to hydrogen production, storage, and fuel cell functionality. Despite the challenges posed by platinum’s cost and scarcity, exploration into alternative metallic nanoparticles showcases promising avenues for sustainable hydrogen technologies. In the realm of hydrogen energy, metallic nanoparticles, particularly platinum nanoparticles, have been extensively researched as catalysts for various reactions. For instance, platinum nanoparticles serve as catalysts in fuel cells due to their effectiveness in the oxygen reduction reaction. Ongoing research explores alternatives like palladium and nickel nanoparticles to address cost and availability concerns [90,91].

### 9.2. Batteries

Nanotechnology and metal oxides can enhance various types of batteries, including lithium-ion (Li-ion), sodium-ion (Na-ion), lithium–sulfur (Li-S), zinc–air, and nickel–metal hydride (NiMH) batteries [92]. These batteries are utilized in a wide range of applications based on their performance attributes. Lithium-ion batteries are widely used in electric vehicles, portable electronics, and grid energy storage due to their high energy and power density. Sodium-ion batteries provide a more cost-effective solution for grid energy storage and electric vehicles. Lithium–sulfur batteries are particularly promising for electric aviation, electric vehicles, and high-energy portable electronics due to their exceptionally high energy density. Zinc–air batteries, known for their high energy density and affordability, are suitable for grid energy storage, electric vehicles, and remote power systems. Nickel–metal hydride batteries are commonly found in hybrid electric vehicles, portable electronics, and backup power systems due to their well-balanced energy and power density, as well as their long cycle life [92]. A representation of the most common battery types is tentatively drawn in (Table 6) and can vary depending on specific battery chemistries and designs.

### 9.3. Supercapacitors

Supercapacitors, or ultracapacitors, are electrochemical capacitors that serve as intermediaries between traditional capacitors and batteries. They can store and release energy at a significantly faster rate than batteries and are capable of withstanding millions of charge and discharge cycles without substantial degradation [99,100,101]. These devices store energy within an electric field, which separates charges between two electrodes immersed in an electrolyte, facilitating ion flow between them. The energy storage mechanism in supercapacitors primarily relies on electrostatic double-layer capacitance and pseudo-capacitance [102,103].

**Figure 9:**
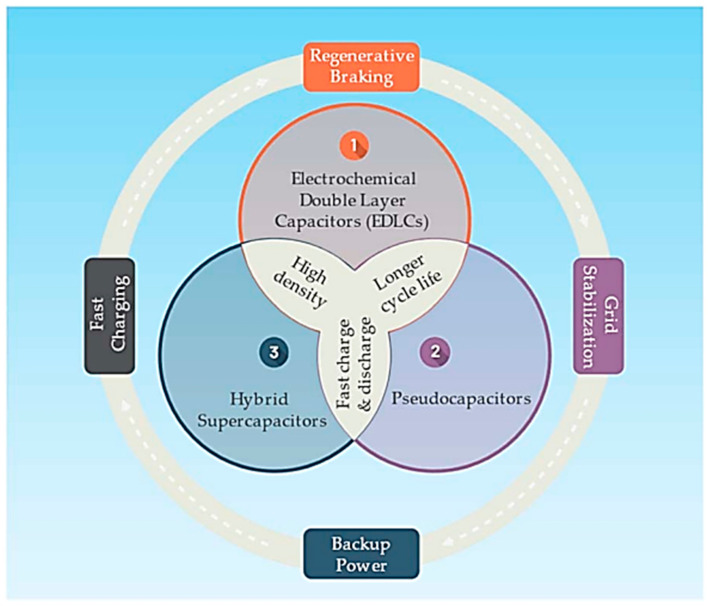
A representation of supercapacitors, including their types, functions, and advantages, with permission from [46].

Supercapacitors are generally classified into three main types: (Figure 9)

- Electrochemical Double-Layer Capacitors (EDLCs): EDLCs store energy in the electric double layer formed at the interface between the electrode and electrolyte, typically using carbon materials with high surface areas as electrodes, such as carbon fiber electrodes [104].
- Pseudo-capacitors: These capacitors store energy through rapid and reversible faradaic redox reactions occurring on the electrode surface, and are usually made of metal oxides or conducting polymers.
- Hybrid supercapacitors: Hybrid supercapacitors combine the characteristics of EDLCs and pseudo-capacitors, utilizing one electrode with high double-layer capacitance and another with high pseudo-capacitance.

Supercapacitors provide higher power density than batteries, allowing for faster charge and discharge cycles. However, they generally have lower energy density, meaning they store less energy per unit volume or mass. Despite this, supercapacitors boast a much longer cycle life, capable of withstanding millions of cycles without significant capacity loss, whereas batteries typically degrade after only a few thousand cycles [105,106]. These features make supercapacitors ideal for applications requiring rapid energy storage and release, such as regenerative braking in electric vehicles, short-term energy storage in grid systems, or backup power solutions. On the other hand, supercapacitors are not suitable for long-term energy storage or applications demanding high energy density, where batteries remain the better option. Future research will focus on the use of metal oxide nanomaterials in applications like photocatalytic water splitting for hydrogen production, solar cells, and thermoelectric materials, with the goal of advancing our understanding and facilitating the development of sustainable energy solutions [107,108]. The most famous application of metal oxides in energy storage systems can be seen in Table 7.

## 10. Nanomaterials Used in Hydrogen Storage

The field of hydrogen storage is witnessing significant advancements with the exploration of a diverse range of nanomaterials, including metal hydrides, complex hydrides, nanotubes, nanofibers, and metal–organic frameworks [109,110,111]. Despite extensive investigations, current storage systems face significant challenges in meeting comprehensive requirements for hydrogen content, release temperature, and reversibility [112]. Recent advancements have focused on nano-structuring techniques, with breakthrough work in 2005 demonstrating nanoconfined borane in mesoporous silica [113] and subsequent studies highlighting the potential of incorporating carbon materials to enhance mechanical stability and thermal management during hydrogen cycling [114]. Despite investigations into promising materials like light metal hydrides (NaH, LiH, MgH_2_) and complex hydrides (alanates, amides, borohydrides), most hydrogen storage systems currently face limitations in hydrogen storage capacity (around 5 wt.%) and struggle with issues related to reaction rates and reversibility. The development of stable crystallites in the 5–10 nm range represents a promising direction for improving hydrogen storage technologies. Carbonaceous materials present an intriguing option for hydrogen storage, characterized by their exceptional adsorption capabilities, high specific surface area, intricate pore microstructure, and low mass density [115]. The fundamental pathway of hydrogen storage in these objects remains incompletely understood, with two primary interaction modes proposed: physisorption takes place through van der Waals forces, while chemisorption involves the formation of more robust chemical bonds.

**Figure 10:**
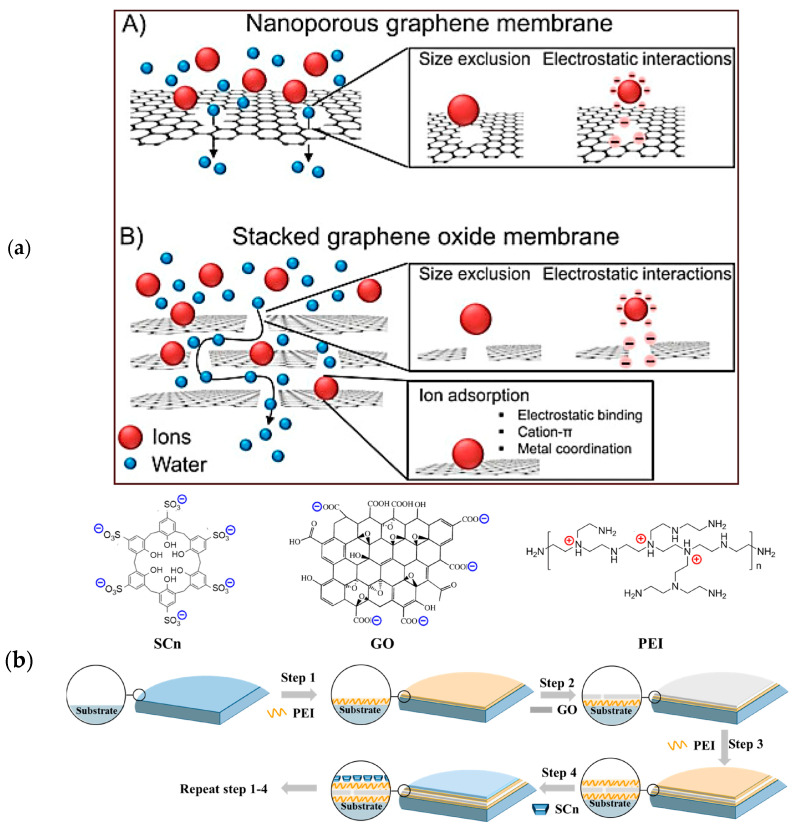
(**a**) Graphene-based ultrathin membranes are advanced materials designed for water separation applications, with permission from [125]. (**b**) Molecular structure of building blocks and schematic illustration of the membrane fabrication process (only the partial membrane structure-adjoining substrate is displayed in the figure [126]).

Typically, physisorption limits the hydrogen-to-carbon ratio to below one hydrogen atom for every two carbon atoms, constraining storage capacity to approximately 4.2 mass percent. Dillon et al.’s groundbreaking research on H_2_ storage in CNTs [116] sparked global interest in carboniferous material research. Subsequent investigations revealed that hydrogen can be physically adsorbed and compressed within carbon structures, with carbon nanotubes demonstrating hydrogen storage densities around 10% of their weight [117]. Fullerenes, characterized by their unique closed-cage molecular structure, have emerged as particularly promising candidates. These carbon allotropes can accommodate H_2_ by hydrogenation of carbon–carbon double bonds, potentially hosting up to 60 hydrogen atoms both internally and externally. The theoretical maximum for fullerene hydrogen storage is exemplified by the C_60_H_60_ isomer, which offers a hydrogen content of approximately 7.7 wt.% [118]. While the fullerene hydride reaction appears reversible at elevated temperatures, complete hydrogen gas release requires temperatures between 823 and 873 K, presenting both opportunities and challenges for practical application. Hydrogen storage technologies are advancing through diverse material approaches, with glass microspheres demonstrating promising potential by achieving storage densities of 14% mass fraction at 25 MPa and 20 kg. H_2_/m^3^ around 62 MPa. Carbon-based sorbents offer versatile storage solutions across multiple structural configurations, including nanotubes, fibers, fullerenes, and activated carbons [119], while advanced crystalline materials like (MOFs = metal–organic frameworks), (ZIFs = zeolitic imidazolate frameworks), and (COFs = covalent organic frameworks) provide additional innovative storage strategies [120,121]. Nanocomposites feature a polyaniline template which can be modified through catalytic doping or the inclusion of nanoscale variants. Research has demonstrated polyaniline’s potential to store hydrogen, with studies reporting hydrogen storage capacities ranging from 6 to 8 wt.% and more recent findings showing uptakes of 1.4–1.7 wt.% [122]. Despite these advances, researchers recognize the critical need to develop innovative reversible materials. Clathrates have emerged as a suggested new material category for hydrogen storage, characterized by hydrogen-bonded water frameworks that can accommodate hydrogen molecules, thereby presenting a potential solution for external hydrogen storage applications. Several carbon-based nanomaterials, including carbon nanodots, fullerenes, graphene, CQDs (carbon quantum dots), and CNTs (carbon nanotubes), have been instrumental in modifying photocatalyst surfaces for hydrogen production [123]. These carbon materials have demonstrated remarkable capabilities in expanding visible-light absorption ranges and facilitating enhanced charge transfer processes. Graphene stands out due to its exceptional properties: superior charge carrier flexibility, expansive surface area, outstanding (electrical and thermal) conductivity, remarkable (physical and chemical) stability, as well as ease of creation [124]. Scientists have extensively explored graphene-based nanocomposites to improve hydrogen production efficiency. Prasad et al. highlighted significant advancements in graphene-based nanocomposites, emphasizing their tailored properties for enhanced energy (storage and environmental) applications. In supercapacitors, the incorporation of graphene with materials such as metal oxides (MOs) and conducting polymers (CPs) effectively addresses challenges such as restacking and agglomeration, leading to improved electrode performance and energy efficiency. Environmental applications show promising results, with graphene composites excelling in heterogeneous catalysis and the elimination of organic dyes from wastewater. The study underscores the potential of these materials to deliver high performance, sustainability, and cost efficiency, advocating for continued innovation to unlock transformative breakthroughs in energy storage and environmental remediation technologies [125]. Ultrathin membranes, such as nonporous graphene membranes and multilayered graphene oxide membranes, are utilized in water separation technologies (Figure 10a). Recent advances have shown that functionalization of graphene plays a major role in the enhancement of hydrogen storage capacity and overall performance. Also, to facilitate water permeation through a GO membrane in the water/ethanol separation process, we introduced SC6 molecules into GO interlayer channels with the assistance of cationic polyelectrolyte polyethyleneimine (PEI) (Figure 10b) The strong electrostatic interactions between GO/PEI and SC6/PEI ensure a stable membrane structure, while the existence of SC6 molecules provides abundant channels for the preferential transport of water molecules, thus leading to a remarkable improvement in both the permeate flux and water/ethanol separation factor [126].

**Figure 11:**
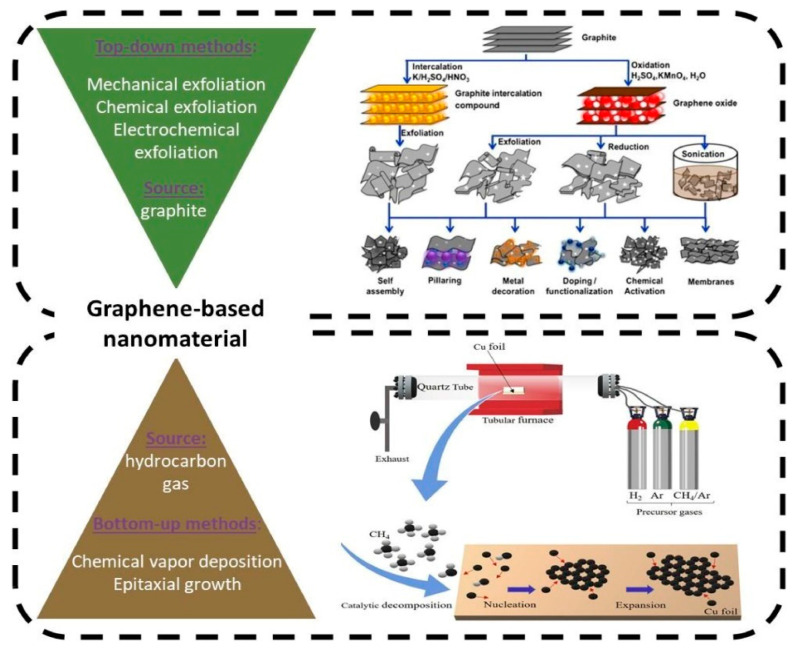
The primary synthetic methods intended for generating graphene-based materials (such as GO, rGO, and nanomaterials) for applications in energy and hydrogen storage, with permission from [127].

**Figure 12:**
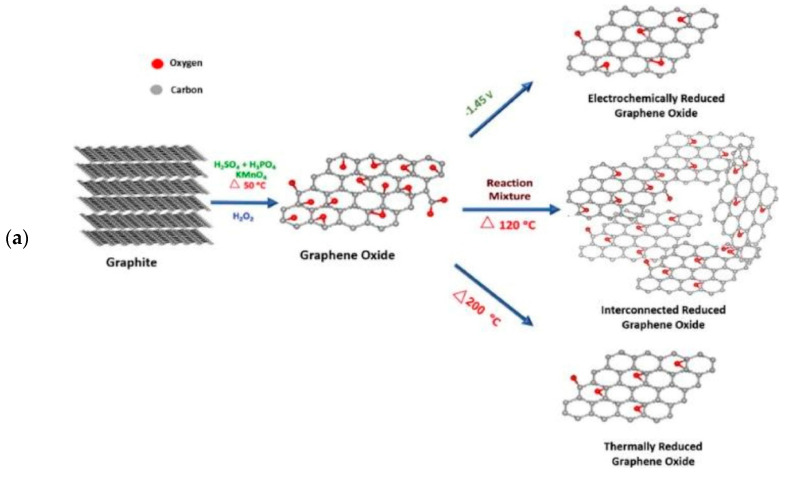

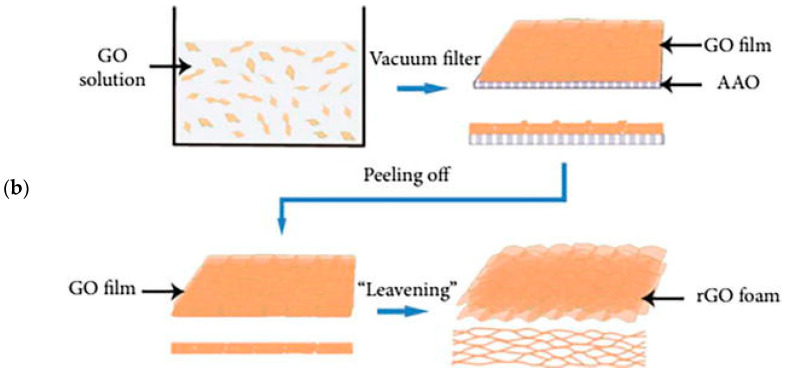
(**a**) Preparation of GO as well as various reduction processes yielding E-rGO, T-rGO, and IC-rGO, with permission from [127]. (**b**) Schematic of the fermentation process to prepare rGO foam [129].

Boateng et al. examined the advancements in graphene-based nanomaterials for energy and hydrogen storage technologies, emphasizing their transformative potential in next-generation energy systems. The study demonstrates that functionalization significantly develops the intrinsic assets of pristine graphene, involving its specific surface area, electric conduction, as well as mechanical steadiness. These enhancements are pivotal, representing energy storage treatments, such as supercapacitors as well as batteries, where functionalized graphene has shown marked improvements in energy density, cyclic stability, and overall efficiency. In hydrogen storage, graphene derivatives, particularly those with hybrid systems or porous architectures, have exhibited promising storage capacities. However, these materials still do not meet the stringent performance benchmarks set by the U.S. Department of Energy. The findings highlight the critical role of advanced synthetic and functionalization strategies in tailoring graphene-based materials for specific applications while addressing scalability and cost-efficiency challenges. The research emphasizes that new and creative solutions are needed to address current challenges in the use of graphene nanomaterials for energy and hydrogen storage [127]. The synthesis of graphene-based materials follows either top-down or bottom-up approaches, as illustrated in Figure 11. Various GO reduction methods are also outlined in Figure 12a [127]. Fan et al. reported (RGO = TiO_2_/reduced graphene oxide) nanocomposites [128], but Hao et al. informed that the integration of titanium dioxide with graphene quantum dots exhibited enhanced photocatalytic hydrogen generation capabilities. Niu et al. performed the reduction of a filtrated GO film by positioning it on the top of a hydrazine monohydrate solution at 90 °C for 10 h (Figure 12b) [129]. The research demonstrated that when combined with TiO_2_, graphene quantum dots effectively function as both electron storage systems and photosensitizers, resulting in improved hydrogen evolution performance. Graphene has emerged as a superior carbon-based material for hydrogen storage applications due to its exceptionally high active surface area and unique chemical characteristics [130]. The material demonstrates effective catalytic properties without requiring metal components, utilizing its beneficial electrochemical and chemical features. Its outstanding physicochemical properties include superior charge carrier mobility, mechanical flexibility, and high thermal conductivity. The distinctive sp^2^ hybridization structure of graphene, which creates its characteristic hexagonal lattice arrangement, provides optimal conditions for hydrogen atom storage [131]. The storage of hydrogen molecules occurs through two fundamental mechanisms: physisorption (physical adsorption) and chemisorption (chemical adsorption).

**Figure 13:**
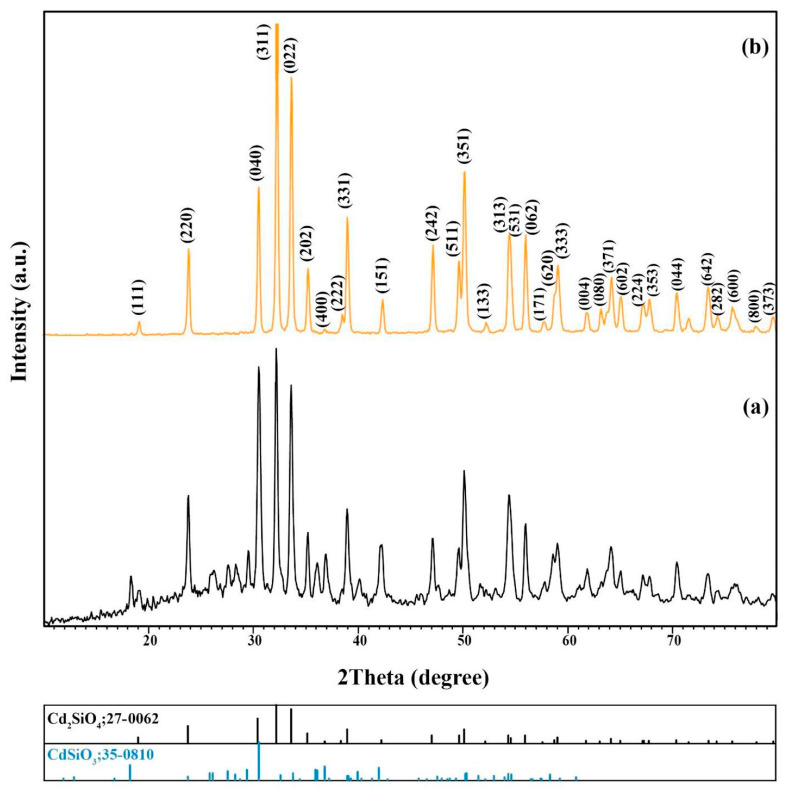
XRD patterns of Cd_2_SiO_4_ nanoparticles synthesized at pH values of (**a**) 7 (sample No. 1) and (**b**) 10 (sample No. 2), with permission from [132].

Masjedi-Arani and colleagues made significant contributions to this field by synthesizing novel Cd_2_SiO_4_/graphene nanocomposites [132]. XRD patterns of Cd_2_SiO_4_ nanoparticles synthesized around pH = 7 (sample No. 1) and pH = 10 (sample No. 2) are shown in Figure 13. Cd_2_SiO_4_ (cadmium silicate) typically has a crystalline structure that provides a high surface area and introduces additional adsorption sites for hydrogen or reactants. Their research demonstrated that Cd_2_SiO_4_ nanoparticles blended into graphene sheets create a composite with enhanced performance. The electrochemical hydrogen storage capacity revealed remarkable improvements: while pure Cd nanoparticles exhibited a storage capacity of 1300 mA hg^−1^, the Cd_2_SiO_4_/graphene nanocomposites achieved an impressive 3300 mA hg^−1^. The marked improvement in storage capacity can be attributed to two key factors: the composite’s optimized charge transport characteristics and its high specific surface area, which collectively yield superior electrochemical hydrogen storage performance compared to pristine graphene structures. Additionally, the material’s inherently low gravimetric density enhances its practicality for hydrogen storage applications.

The enhancement in materials containing Cd_2_SiO_4_ compared to pure graphene in certain applications (e.g., hydrogen storage, energy storage, catalysis, etc.) arises from the synergistic interaction between Cd_2_SiO_4_ and graphene. Here is an overview of the mechanism behind this enhancement [133,134]:

1. **Structural and Synergistic Effects**

- Cd_2_SiO_4_ (cadmium silicate) typically has a crystalline structure that provides a high surface area and introduces additional adsorption sites for hydrogen or reactants.
- When combined with graphene, the 2D structure of graphene provides an extensive conductive network, ensuring rapid charge or energy transfer.
- The hybrid structure of graphene and Cd_2_SiO_4_ creates synergistic effects, where graphene stabilizes the dispersion of Cd_2_SiO_4_ nanoparticles and prevents their agglomeration, while Cd_2_SiO_4_ enhances the overall functionality by contributing active sites.

2. **Enhanced Adsorption and Storage Capacity**

- Cd_2_SiO_4_ contributes to an increased number of reactive sites for gas adsorption (e.g., hydrogen). These sites arise from its unique crystal structure and surface properties.
- Graphene further enhances this by providing a high surface area, ensuring better gas diffusion and exposure to active sites.

3. **Improved Electronic Properties**

- Graphene has excellent electrical conductivity, while Cd_2_SiO_4_ typically acts as a semiconductor. When combined:

○ The heterojunction between graphene and Cd_2_SiO_4_ enhances charge separation and transport.
○ In catalysis or hydrogen storage, this can reduce energy barriers and improve reaction kinetics, leading to better performance than pure graphene.

4. **Defect Engineering**

- The introduction of Cd_2_SiO_4_ into the graphene matrix introduces defects and strain in the graphene lattice.
- These defects act as nucleation points for hydrogen adsorption or catalytic activity, significantly improving the storage capacity and reactivity of the material.

5. **Thermodynamic and Kinetic Effects**

- Cd_2_SiO_4_ can lower the energy barrier for processes such as hydrogen adsorption/desorption or other catalytic reactions, making the system more efficient.
- Graphene, with its excellent thermal and mechanical stability, provides structural support and ensures that the material can withstand multiple cycles without significant degradation.

6. **Surface Chemistry**

- Cd_2_SiO_4_ has polar surfaces that can enhance interactions with hydrogen or other reactants through chemisorption or physisorption.
- Graphene, being chemically inert, benefits from the active chemical nature of Cd_2_SiO_4_, while maintaining a conductive and stable scaffold.

**Application-Specific Mechanism**

The exact mechanism of enhancement can vary depending on the application:

- Hydrogen Storage: Cd_2_SiO_4_ provides additional sites for hydrogen adsorption, and graphene ensures rapid hydrogen diffusion and release.
- Catalysis: The combination of Cd_2_SiO_4_ and graphene provides a balance of active sites and electron mobility, boosting catalytic efficiency.
- Energy Storage: In batteries or supercapacitors, Cd_2_SiO_4_ contributes to ion storage, while graphene enhances conductivity and rate performance.

**Figure 14:**
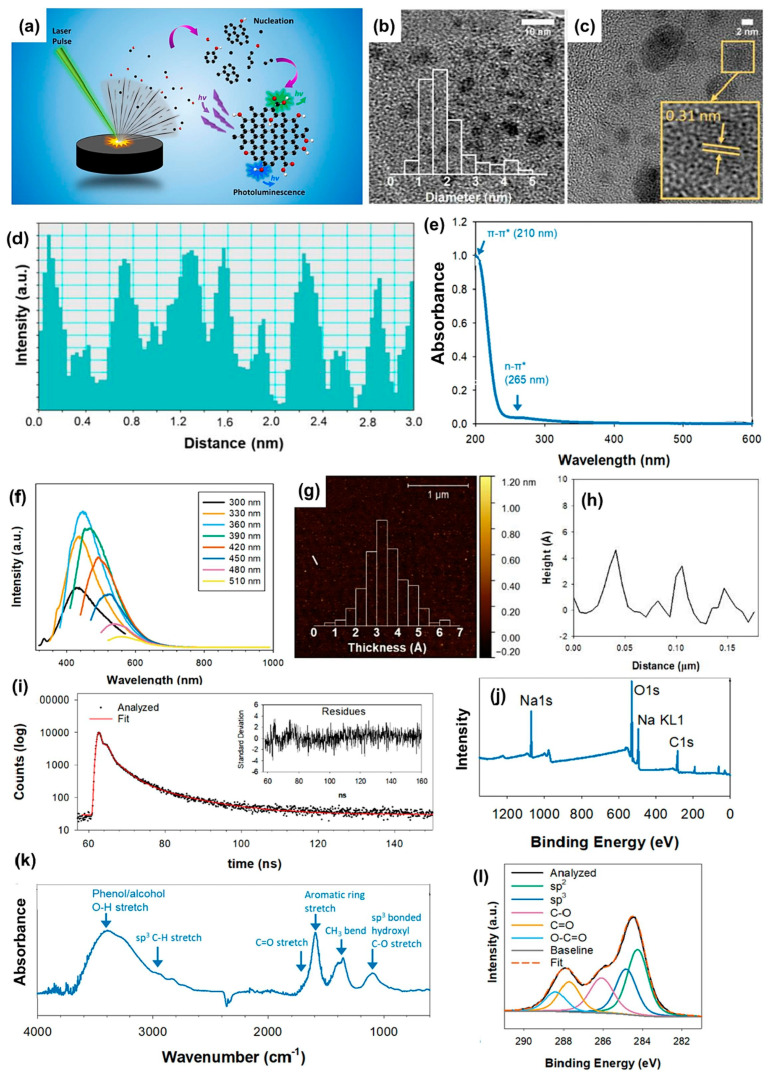
(**a**). Graphical representation of laser ablation synthesis. (**b**). TEM images. (**c**). Lattice profile of GQD. (**d**). Diameter distribution. (**e**). UV–Vis absorbance. (**f**). Photoluminescence spectra. (**g**). AFM images. (**h**) Corresponding height profiles. (**i**). TCSPC decay curves. (**j**) Survey XPS spectra. (**k**). Normalized FTIR. (**l**) high-resolution C1s spectra of GQD synthesized from laser ablation, with permission from [135].

Dananjaya et al. provide a comprehensive review that discusses the recent progress in the synthesis, properties, applications, 3D printing, and machine learning of graphene quantum dots (GQDs) in polymer composites. The review explores various synthesis methods, emphasizing size control and surface functionalization of GQDs. It examines the unique electronic structure, tunable bandgap, and optical properties of GQDs, while discussing strategies for incorporating GQDs into polymer matrices and their effects on mechanical, electrical, thermal, and optical properties. Applications of GQD-based polymer composites in optoelectronics, energy storage, sensors, and biomedical devices are also reviewed. The challenges and future prospects of GQD-based composites are addressed, aiming to provide researchers with a comprehensive understanding of advancements in related fields. Moreover, the article explores new developments in 3D printing technology that benefit from composite materials loaded with GQDs, a promising class of materials with broad potential applications. Additionally, this review delves into the emerging role of machine learning techniques in optimizing GQD–polymer composite materials and examines how artificial intelligence and data-driven approaches revolutionize the design and characterization of these nanocomposites. These advances enable researchers to navigate the vast parameter space efficiently to achieve the desired properties. The review ultimately aims to connect individual aspects of synthesis, properties, applications, 3D printing, and machine learning of GQDs in polymer nanocomposites, providing a cohesive and comprehensive resource for readers. Carbon nano-onions (nCNOs) were chemically oxidized (CO) before being pulsed laser-abated (LA) in one step in deionized water to create graphene quantum dots. According to the photoluminescence (PL) spectra, the impacts of both particle sizes and surface functional groups account for the LA-GQDs’ blue-shifted emission compared to the CO-GQDs’. While the LA-GQDs have an average diameter of 1.8(6) nm and a thickness similar to one layer of graphene, the CO-GQDs have an average diameter of 4.1(8) nm and a thickness equivalent to two or three graphene layers, as presented in Figure 14 [135].

**Figure 15:**
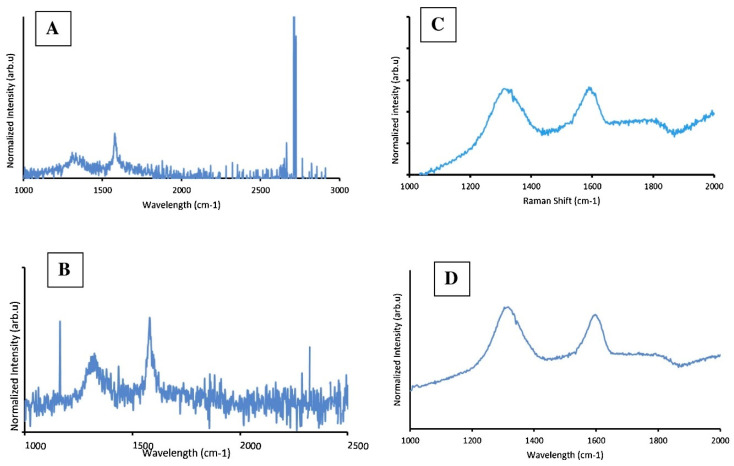
Raman spectra of sample (**A**): graphite flakes, (**B**): GO, (**C**): NH_3_-reduced graphene, and (**D**): ascorbic acid-reduced graphene, with permission from [136].

**Figure 16:**
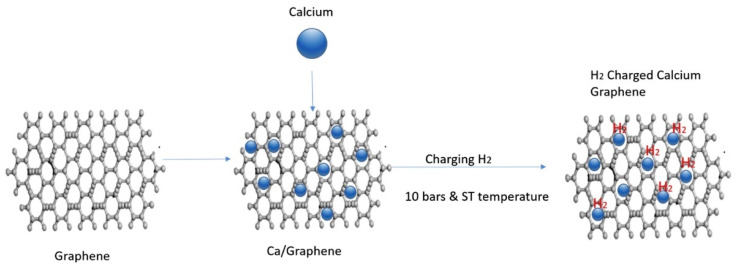
Ca/graphene hydrogen storage mechanism, with permission from [136].

Ngqalakwezi and colleagues developed graphene nanocomposites using a modified Tours method [136]. The spectroscopic analysis presented in (Figure 15) illustrates the comparative spectra of four distinct materials: graphite flakes, graphite oxide, ammonia-reduced graphene, and L-ascorbic acid-reduced graphene, each demonstrating progressive enhancement in hydrogen uptake capacity. The interaction mechanism involves calcium atoms or clusters that form stable attachments to the carbon nanostructure. These calcium sites facilitate hydrogen molecule binding without molecular dissociation through two possible mechanisms: the Kubas interaction or directional polarization. This results in significantly stronger binding energies, approaching 1 eV per H_2_ molecule, which represents a substantial improvement over unmodified graphene’s binding energy of 0.07 eV per H_2_ molecule. The schematic representation of this mechanistic process is illustrated in Figure 16 [136]. Their research showed hydrogen storage capacities increasing to 4.98 wt.% in graphene and 3.99 wt.% in Ca/graphene nanocomposites, with ammonia, in addition, further enhancing storage capabilities. The same research group explored reduced graphene oxide–magnesium composites [137], achieving remarkable hydrogen storage performance of 6.5 wt.% and 0.105 kg hydrogen per liter in the total composite.

**Figure 17:**
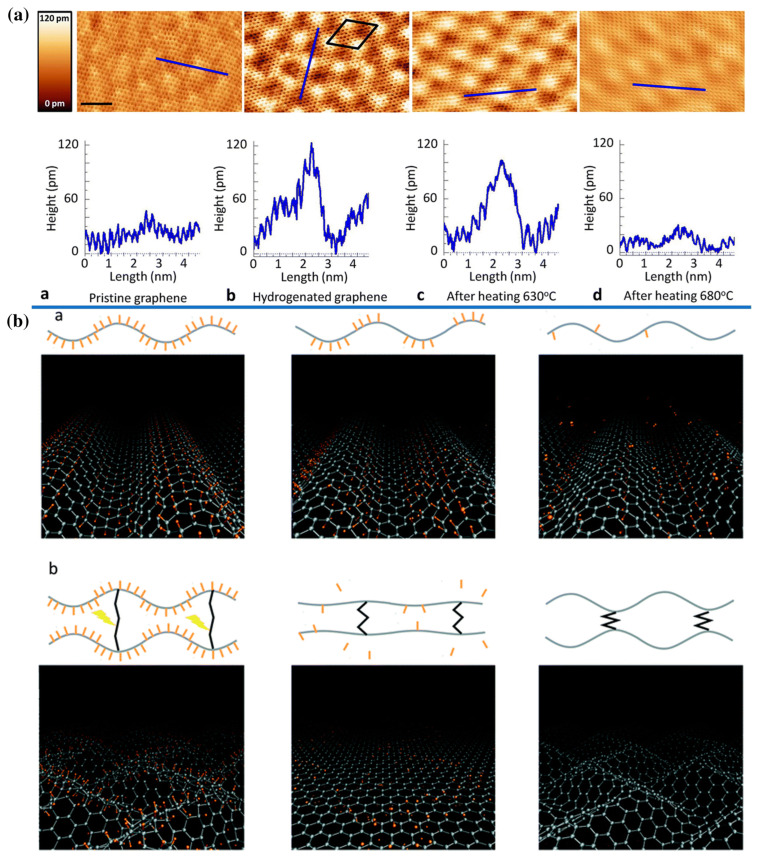
(**a**) An STM image of pristine graphene with a cross-sectional view below. After 5 s of exposure to atomic hydrogen, the STM image showed low hydrogen coverage and significant corrugation due to C–H bond formation on the convex areas of the graphene surface. Annealing the sample at 630 °C and 680 °C resulted in complete hydrogen desorption at 680 °C. (**b**) Possible methods for hydrogen desorption include mechanical distortion using piezoelectric substrates and altering the size of intercalants with external stimuli, with permission from [143].

**Scheme 2:**
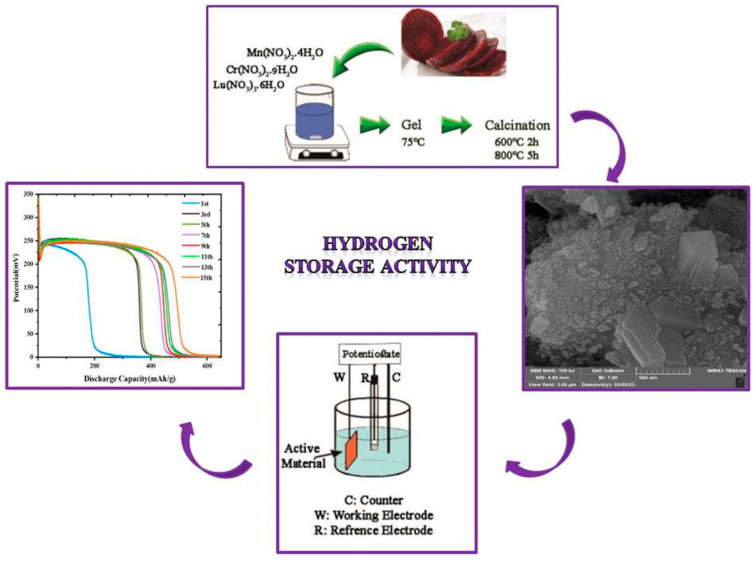
A diagram showing how LCMO functions as a material for storing hydrogen, with permission from [173].

Ravi and Grace expanded this research by fabricating MnFe_2_O_4_/G and ZnFe_2_O_4_/G nanocomposites for hydrogen evolution [138]. Other notable developments included metal-decorated Ni/graphene-like materials (GLM) for hydrogen applications [139]; ZnAl_2_O_4_/graphene nanocomposites synthesized using green tea and olive leaf extracts, achieving 67.5% coulombic efficiency and 3100 mAh g^−1^ discharge capacity [140]; and 3D-graphene materials doped with TiO_2_ through electrostatic assembly, demonstrating enhanced storage properties with a pore volume of 0.41 cm^3^g^−1^ and surface area of 705 m^2^g^−1^ [141]. Particularly impressive were the Pd/graphene nanocomposites, which exhibited H_2_ storage capacities of 8.67 wt.% (around 1% Pd and 60 bar pressure) and 7.16 wt.% (uptake capacity) [142]. Jain and Kanda Subramanian recently provided a comprehensive review of functionalized graphene’s role in hydrogen energy storage [143], highlighting the ongoing scientific interest in this field. An STM image of pristine graphene is shown below, with a cross-sectional view. After 5 s of exposure to atomic hydrogen, the STM image revealed low hydrogen coverage and noticeable surface corrugation due to the formation of C–H bonds on the convex regions of the graphene surface. Annealing the sample at 630 °C and 680 °C led to complete hydrogen desorption at 680 °C, as shown in Figure 17a. Potential methods for hydrogen desorption include mechanical distortion using piezoelectric substrates and adjusting the size of intercalants through external stimuli as shown in Figure 17b [143]. A review examining graphene and its composite materials as potential solutions for hydrogen storage applications is shown in Table 8. Mahdi et al. introduced Lu_2_CrMnO_6_ (LCMO) nanostructures as a novel hydrogen storage material synthesized via an eco-friendly sol–gel method using beetroot juice. Using multiple characterization techniques, researchers found that LCMO-10 (made with 10 mL beetroot juice) achieved superior hydrogen storage capacity compared to LCMO-5, reaching 570 mAh/g by the 15th cycle. The enhanced performance was attributed to LCMO-10’s morphology providing more active sites. The results demonstrate LCMO-10’s potential as an effective electrode material for electrochemical hydrogen storage through physisorption and redox processes, as shown in Scheme 2.

**Table 8:** An overview examining graphene and its composite materials as potential solutions for hydrogen storage applications.

**Graphene Nanocomposite**	**Material Composition** **Graphene (G)**	**H_2_ Storage Capacity (wt.%)**	**Reference**
	rGO/transition metal catalysts	6–8	[130]
	G with porous carbon	7	[130]
	GO/(MOFs)	4	[144]
	G/Al	5.13	[145]
	rGO with MoS_2_	5.8	[146]
	G/Li	12.8	[147]
	r GO/Mg	6.5	[148]
	GO/Ti	4.9	[149]
	G/Ca	8.4	[150]
	G/Li/B	10.7	[151]
	Pt/Pd/G	0.16	[152]
	Cu-BTC/G	3.58	[153]
	Be/B/Doped G	15.1	[154]
	N-Doped G	7.23	[155]
	Li/Fullerene/G	5	[156]
	Hierarchical G	4.01	[157]
	G Nanosheets	1.2	[158]
	GO/MWCNT	2.6	[159]
	N/Pd/Decorated G	2.1	[160]
	Ni & B/G	4.4	[161]
	GO/MoS_2_, Ni-based catalysts)	7	[162]
	GO/(CuFe_2_O_4_)	3.3	[163]
	N-doped G-Ni (NPs) (Ni@NC)	6.5	[164]
	Ni_6_MnO_8_@rGO	6.6	[165]
	Ni-Nb/rGO	4.5 wt.%	[166]
	C_3_N_4_–MoS_2_–Ni(OH)_2_	2.79 wt.%	[167]
	MWCNTs–CoNiFe_2_O_4_	1.65 and 1.12 wt.%	[168]
	Mg/MgH_2_	7.6 wt.%	[169]
	Yb_2_O_3_@C	3 wt.%	[170]
	Mg-Mg_2_Ni/C	6 wt.%	[171]
	Mg_96_La_3_Ni–Eu_2_O_3_@C	2 wt.%	[172]
	Mg–C–G	3.72 wt.%	[173]

Recent advances have shown that functionalization of graphene plays a major role in the enhancement of hydrogen storage capacity and overall performance. Some of them are listed in Table 9, along with the functionalization method, type of storage medium, and storage capacity, in order to better represent the state of the art.

### The Merits of the Solid-State Approach for H_2_ Storage

The merits of the solid-state approach for hydrogen (H_2_) storage compared to conventional gas and liquid storage methods are as follows.

1. **Enhanced Safety**
  Solid-state hydrogen storage operates at lower pressures (typically below 10 MPa), reducing risks associated with high-pressure tanks (350–700 bar) used in gas storage.
  It minimizes explosion hazards, hydrogen leakage, and embrittlement issues common in high-pressure cylinders and cryogenic tanks.
  Hydrogen is stored in a chemically bound or absorbed form, making it less prone to accidental release.
2. **Higher Storage Density**
  Solid-state materials can achieve higher volumetric hydrogen densities than compressed gas.
  Example: Metal hydrides (e.g., MgH_2_, LaNi_5_H_6_) can store hydrogen at densities greater than 150 kg H_2_/m^3^, exceeding the storage capacity of compressed hydrogen at 700 bar.
3. **Reversible Hydrogen Absorption and Release**
  Many materials allow on-demand hydrogen release at moderate temperatures and pressures.
  Example: Sodium alanate (NaAlH_4_) releases H_2_ at temperatures below 200 °C with catalytic doping.
  It is suitable for proton exchange membrane fuel cells (PEMFCs) and other hydrogen-based energy systems.
4. **Energy Efficiency and Sustainability**
  Solid-state storage avoids the energy-intensive liquefaction (cryogenic cooling at −253 °C) and high-pressure compression processes, reducing energy costs and carbon footprint.
  Many solid-state materials (e.g., borohydrides, metal–organic frameworks (MOFs)) can be recycled and reused, making them more sustainable.
5. **Compact and Lightweight Storage Solutions**
  Porous materials like MOFs, covalent organic frameworks (COFs), and porous carbons provide high surface areas and tunable pore structures, allowing for efficient hydrogen storage in a compact form.
  Example: Some MOFs achieve gravimetric hydrogen storage capacities above 6 wt.%, making them competitive with conventional methods.
6. **Compatibility with Fuel Cells and Portable Applications**
  Solid-state hydrogen storage materials integrate well with fuel cells, enabling direct hydrogen supply for vehicles, portable electronics, and industrial applications.
  Metal hydrides and chemical hydrides are being explored for hydrogen-powered cars, drones, and backup power systems.
7. **Scalability for Industrial and Mobile Applications**
  Certain metal hydrides (e.g., Ti-doped NaAlH_4_, LaNi_5_H_6_) are already commercially viable for hydrogen storage in stationary and mobile applications. They have the potential for large-scale use in hydrogen-powered transportation, aerospace, and grid energy storage.
  Overall, the solid-state approach offers a safe, high-density, and energy-efficient solution for hydrogen storage, making it a strong candidate for future hydrogen-based energy systems.

## 11. Challenges of Using Nanomaterials in Energy Storage and Hydrogen Production

The use of nanomaterials in energy storage and hydrogen production offers exciting possibilities, but it also faces several challenges. Below, we outline the challenges in these fields:

1. *Synthesis and Scalability*
  - **Complex Synthesis Processes**: Producing nanomaterials often requires sophisticated techniques, such as chemical vapor deposition, hydrothermal synthesis, or electrochemical methods, which can be expensive and time-consuming.
  - **Scalability**: Translating laboratory-scale production to industrial-scale manufacturing remains a significant hurdle due to cost, consistency, and quality control issues.
2. *Stability and Durability*
  - **Material Degradation**: Nanomaterials can undergo degradation during prolonged use due to oxidation, agglomeration, or chemical reactions in harsh operational environments.
  - **Performance Decay**: Stability over multiple cycles in batteries or during hydrogen evolution reactions is often limited.
3. *Cost Concerns*
  - **High Cost of Raw Materials**: Precious metals like platinum are often used in catalysts for hydrogen production, driving up costs.
  - **Processing Costs**: Nanomaterial production and integration into devices require high precision and expensive equipment.
4. *Safety and Environmental Impact*
  - **Toxicity**: Some nanomaterials, such as metal oxides or quantum dots, can be toxic, posing risks during synthesis, usage, and disposal.
  - **Environmental Concerns**: Life cycle environmental impacts, including the energy-intensive production process, need better assessment.
5. *Integration Challenges*
  - **Compatibility**: Integrating nanomaterials into existing energy storage and hydrogen production systems can be difficult due to mismatches in material properties or fabrication methods.
  - **Scaling Devices**: Efficiently embedding nanomaterials into large-scale energy storage or hydrogen production systems without sacrificing performance remains challenging.

## 12. Future Prospects

The global transition to renewable energy sources demands innovative solutions to address challenges in energy storage and hydrogen production. Nanomaterials have emerged as a game-changing technology in these domains due to their unique physical, chemical, and electronic properties. Their high surface area, tunable characteristics, and superior catalytic activities make them promising candidates for revolutionizing energy technologies. The future prospects of nanomaterials in energy storage and hydrogen production highlight their potential to transform these sectors sustainably and efficiently as follows.

### 12.1. Advancements in Energy Storage Technologies

Energy storage systems, such as batteries and supercapacitors, are pivotal for stabilizing renewable energy systems. Nanomaterials offer significant opportunities to enhance the performance of these technologies:

- **High-Energy-Density Batteries**: Nanostructured materials, such as silicon nanowires and lithium–metal nanoparticles, can significantly increase the energy density of batteries. Silicon-based anodes, for instance, offer a theoretical capacity ten times greater than conventional graphite anodes, making them ideal for next-generation lithium-ion batteries.
- **Improved Cycle Life**: One major challenge in energy storage is maintaining stability over multiple charge–discharge cycles. Nanomaterials like graphene and carbon nanotubes can improve electrode stability by accommodating volumetric changes and mitigating structural degradation during cycling.
- **Fast Charging and High Power**: The incorporation of nanostructured materials into supercapacitors enhances ion diffusion and conductivity, enabling faster charging and discharging cycles. This can lead to the development of hybrid energy storage devices that combine the high energy density of batteries with the rapid charge–discharge capabilities of supercapacitors.
- **Solid-State Batteries**: Solid electrolytes augmented with nanomaterials provide enhanced ionic conductivity and mechanical stability, paving the way for safer and more energy-dense solid-state batteries.

### 12.2. Innovations in Hydrogen Production

Hydrogen is increasingly recognized as a clean energy carrier, but efficient and sustainable production methods are critical for its widespread adoption. Nanomaterials offer revolutionary possibilities in this area:

- **Enhanced Catalysts for Water Splitting**: Traditional catalysts, such as platinum, are expensive and scarce. Nanomaterials, including transition metal dichalcogenides (e.g., MoS_2_) and perovskite nanostructures, provide high catalytic efficiency at a fraction of the cost. Single-atom catalysts further enhance activity by maximizing active sites.
- **Photoelectrochemical (PEC) Hydrogen Production**: Semiconductor nanomaterials like titanium dioxide (TiO_2_) and cadmium sulfide (CdS) are being optimized for PEC water splitting. By tailoring their band gaps and surface properties, these materials can achieve higher solar-to-hydrogen conversion efficiencies.
- **Electrochemical Hydrogen Evolution**: Nanostructured electrodes improve the kinetics of the hydrogen evolution reaction (HER) by reducing overpotentials. For example, graphene-supported catalysts provide high conductivity and durability.
- **Thermocatalytic Processes**: In high-temperature environments, nanomaterials such as cerium oxide (CeO_2_) enable efficient hydrogen production from methane reforming or biomass-derived feedstocks, contributing to green hydrogen production.

### 12.3. Integration with Renewable Energy Systems

Nanomaterials can play a crucial role in integrating energy storage and hydrogen production with renewable energy sources such as solar and wind power:

- **Energy Storage for Grids**: Nanomaterials can enhance large-scale battery systems used to store excess energy generated by intermittent renewable sources. This ensures grid stability and reliability.
- **Hydrogen Production Coupled with Renewables**: Electrolyzers incorporating nanocatalysts can be powered by solar or wind energy to produce green hydrogen. Advanced nanomaterials help reduce the energy input required for splitting water, making this process more cost-effective.
- **Hybrid Energy Systems**: Combining nanomaterial-based storage devices with hydrogen production systems creates synergistic solutions. For instance, excess electricity can be stored in batteries or converted to hydrogen for later use, enhancing overall energy system efficiency.

### 12.4. Sustainability and Green Innovations

The future development of nanomaterials must align with sustainability principles to minimize environmental impacts:

- **Green Synthesis Methods**: Eco-friendly synthesis approaches, such as using plant-based precursors or low-energy chemical processes, can reduce the environmental footprint of nanomaterials.
- **Recyclability**: Designing nanomaterials for easy recycling or repurposing can extend their life cycle and reduce waste.
- **Earth-Abundant Materials**: Transitioning from precious metals to earth-abundant alternatives like iron, cobalt, or nickel in nanomaterial synthesis can address resource scarcity and cost issues.

### 12.5. Leveraging Artificial Intelligence (AI) and Machine Learning

AI and machine learning can accelerate the development and optimization of nanomaterials for energy applications:

- **Material Discovery**: Predictive modeling can identify novel nanomaterials with desired properties, reducing the time and cost of experimental research.
- **Performance Optimization**: Machine-learning algorithms can optimize the design and operation of devices integrating nanomaterials, ensuring maximum efficiency.
- **Life cycle Assessment**: AI tools can evaluate the environmental and economic impacts of nanomaterials, guiding sustainable development practices.

## 13. Conclusions

This comprehensive analysis elucidates the revolutionary impact of nanomaterials on hydrogen energy technology advancement, with particular emphasis on storage mechanisms, production methodologies, and transport systems. Notably, functionalization approaches, specifically those incorporating graphene-based derivatives, have demonstrated exceptional potential in augmenting hydrogen storage capacity and catalytic performance. Contemporary innovations, including three-dimensional hierarchical porous graphene architectures, metal oxide catalytic systems, and hybrid nano-structural configurations, exemplify the remarkable adaptability of nanomaterials in addressing fundamental challenges within the hydrogen economy paradigm. Surface modification and functionalization methodologies, encompassing heteroatom incorporation and defect engineering strategies, have proven instrumental in optimizing hydrogen adsorption kinetics and storage efficiency. Recent developments in photocatalytic and electrocatalytic systems demonstrate nanomaterials’ capacity to enhance hydrogen evolution reactions while maintaining economic viability and scalability parameters. The successful integration of these advanced materials into practical energy storage and conversion systems presents unprecedented opportunities for revolutionizing renewable energy infrastructure. To advance the field further, researchers must address critical challenges in scalability, economic feasibility, and performance optimization to maximize nanomaterial utilization in hydrogen economy applications. Future investigations should focus on developing multifunctional nano-structural architectures, biomimetic designs, and artificial intelligence-optimized systems to overcome existing technological limitations. These advancements are crucial for establishing a sustainable hydrogen-based energy economy and achieving global sustainability objectives.

## Figures and Tables

**Table 1:** Approaches to improve the catalytic performance of HER electrocatalysts [46].

No.	Strategy	Definition	Advantages	Application
1	Strain Engineering	Applying mechanical strain to catalyst materials	Enhanced activity and selectivity, longer-lasting stability	H_2_O splitting, H_2_ fuel cells, HER
2	Crystallinity Engineering	Modifying the crystalline structure of catalyst materials	Better catalytic performance, selectivity, and durability	
3	Doping	Adding foreign atoms to catalyst materials	Improved efficiency, precision, and durability	
4	Valence Regulation	Adjusting the valence states of catalyst components	Improved efficiency, precision, and stability	
5	Catalyst Design	Developing catalysts to boost hydrogen evolution reaction (HER) efficiency	Greater HER efficiency, extended catalyst lifespan	H_2_O splitting, H_2_ fuel cells
6	Nano structuring	Designing catalysts with nanostructures	Increased surface area, higher catalytic activity, better stability	
7	Electrolyte Optimization	Refining electrolyte composition and properties for better performance	Enhanced HER efficiency, improved stability, cost reduction	
8	Interface Engineering	Adjusting catalyst–electrolyte interface characteristics	Improved activity, selectivity, and durability	
9	Co-Catalyst Integration	Incorporating secondary catalysts into the system	Boosted catalytic performance and stability	
10	Light Absorption Enhancement	Increasing the material’s ability to absorb light	Greater photoconversion efficiency, enhanced stability	Photo electro-chemical, H_2_O splitting
11	Atomic-Scale Synergy	Optimizing interactions between individual atoms or molecules to improve performance	Enhanced efficiency, precision, and durability	Electrocatalysis, photocatalysis
12	Phase-Structure Engineering	Regulating crystal structure and phase to enhance catalyst performance	Improved efficiency, precision, and durability	
13	Surface Reconstruction	Altering the surface structure and composition of catalysts	Enhanced catalytic performance and stability	
14	Oxygen Vacancy Engineering	Creating oxygen vacancies in metal oxides to enhance performance	Higher activity and improved durability	
15	Hybridization	Combining different materials to form hybrids with enhanced properties	Better activity, stability, and selectivity	Electrocatalysis, photocatalysis, energy storage
16	Morphology Engineering	Customizing the size, shape, and surface characteristics of catalyst particles	Improved activity, selectivity, and charge/mass transfer	

**Table 2:** Analysis of key performance metrics for various metal oxides in the hydrogen evolution reaction (HER).

Metal Oxide		Overpotential (mV)	Current Density (mA cm^−2^)	Stability (Hours)	TOF (s^−1^)	Tafel Slope (mV dec^−1^)	Refs.
Molybdenum oxide	MoO_x_	150	10	100	0.5	60	[43,44,45]
Iron oxide	FeO_x_	140	12	95	0.6	58	
Copper oxide	CuO_x_	135	11	90	0.55	59	
Zinc oxide	ZnO_x_	145	9	105	0.52	57	
Titanium oxide	TiO_x_	138	11.5	98	0.58	56	
Tungsten oxide	WO_x_	142	10.5	96	0.57	58	
Nickel oxide	NiO_x_	137	12.5	94	0.59	55	
Cobalt oxide	CoO_x_	139	11.8	97	0.56	54	

**Table 3:** The various hydrogen generation processes.

Method	Key Materials	Reaction	Challenges
Electrochemical	Platinum, iridium, alkaline electrolyzers	2H_2_O → 2H_2_ + O_2_	High energy consumption, electrode material cost
Photoelectrochemical	TiO_2_, BiVO_4_, Cu_2_O	2H_2_O → 2H_2_ + O_2_ (light-powered)	Efficiency limitations, recombination losses
Photocatalytic	TiO_2_, MoS_2_, graphene, CdS	2H_2_O → 2H_2_ + O_2_ (light-driven)	Stability, poor charge separation
Electrophotochemical	TiO_2_-based composites, hybrid photoelectrodes	2H_2_O → 2H_2_ + O_2_ (electrochemical + photonic)	Control of inputs for optimal efficiency

**Table 4:** Summary of photocatalytic reactions involving H_2_ evolution, CO_2_ reduction, and photocatalytic organic synthesis using various MOFs-based catalysts, with permission from [68].

Photocatalyst	Light Source (Sacrificial Agent)	Photoactivity	Stability Evidence
Photocatalytic H_2_ evolution			
Pt@MOF-1 or Pt@MOF-2	450 W Xe-lamp (TEA), Visible	3400 or 7000 TON	reuse, leaching
H2TCPP[AlOH]_2_(DMF3-(H_2_O)_2_)	300 W Xe lamp (EDTA), Visible	200 μmol. g^−1^ h^−1^	SEM, XRD
{[Ln_2_Cu_5_(OH)_2_(pydc)_6_(H_2_O)_8_]⋅I_8_} (Ln: Tb)	500 W Hg lamp (aq. CH_3_OH), UV	2105 μmol h^−1^ g^−1^	reuse, IR, XRD
MOF-253-Pt	300 W Xe lamp (TEOA), Visible	3000 μmol	reuse
Pt/NH_2_-UiO-66	200 W Xe-doped Hg lamp (CH_3_OH)	2.8 mL	–
NH2-UiO-66(Zr/Ti)-120-16 days	300 W Xe lamp (CH_3_CN and TEOA)	3.5 mmol mol^−1^	–
RhB/UiO-66(Zr)-100*	300 W Xe lamp (TEOA)	33.9 μmol. g^−1^ h^−1^	XRD
Pt-UiO-66-30	300 W xenon lamp (20 % CH_3_OH)	37 μmol	reuse, ICP-AES
Pt/Ti-MOF-Ru(tpy)_2_	500 W Xe lamp (TEOA)	5.1 μmol	reuse, XRD
Pt/[Cu(en)_2_]_4_[PNb_12_O_40_(VO)_6_]⋅(OH)_5_⋅8 H_2_O	125 W Hg lamp (aq.CH_3_OH)	44.35 μmol. g^−1^ h^−1^	XRD
Fe_2_O_3_@TiO_2_/Pt	Xe lamp (TEA)	0.8 μmol	EDS, TEM, XRD, reuse
Pt@CdS/MIL-101(Cr)	300 W Xe lamp (lactic acid)	150 μmol h^−1^	reuse, XRD
UiO-66/CdS/rGO	300 W Xe lamp (aq. 0.1 M Na_2_S and Na_2_SO_3_)	105 μmol h^−1^	XRD
Pt/NH_2_-MIL-125(Ti)	Xe lamp (TEOA)	33 μmol	reuse, BET, XRD
Pt(1.5)/NH_2_-MIL-125(Ti)	500 W Xe lamp (TEOA)	15.5 μmol	reuse
Pt/MIL-125(Ti)	Xe lamp (λ = 320–780 nm) (TEOA)	38.68 μmol	–
Co@NH_2_-MIL-125(Ti)	500 W Xe/Hg lamp	37 μmol	reuse
Photocatalytic CO_2_ reduction			
NH_2_-MIL-125(Ti)	Xe lamp (CH_3_CN/TEOA)	8.14 μmol HCOO^−^	XRD, BET, TGA, IR, Raman
Au/NH_2_-MIL-125(Ti) or Pt/NH_2_-MIL-125(Ti)	300 W Xe lamp (TEOA)	9.06 μmol or 12.96 μmol HCOO^−^	–
NH_2_-UiO-66	500 W Xe lamp (TEOA)	13.2 μmol HCOO^−^	XRD, IR, BET
NH_2_-UiO-66(Zr/Ti)-120-16 days	300 W Xe lamp (CH_3_CN and TEOA)	5.8 mmol mol^−1^	XRD, BET
Zr_4.3_Ti_1.7_O_4_(OH)_4_(C_8_H_7_O_4_N)_5.17_(C_8_H_8_O_4_N_2_)_0.83_	300 W Xe lamp (CH_3_CN and TEOA)	33 μmol HCOO^−^	Reuse
MIL-101(Fe) or NH_2_-MIL-101(Fe)	300 W Xe lamp (CH_3_CN and TEOA)	59 or 178 μmol HCOO^−^	reuse, XRD, IR, TGA, BET
Cu porphyrin (5,10,15,20-tetrakis(4-carboxyphenyl)	300 W Xe lamp (TEA)	262.6 ppm g^−1^ h^−1^ CH_3_OH	–
MOF-253-Ru(CO)_2_Cl_2_	Xe lamp (CH_3_CN and TEOA)	0.67 μmol HCOO^−^	XRD
Cp*Rh@UiO-67	300 W Xe arc lamp (CH_3_CN and TEOA)	47 TON	Reuse
Cu_3_(BTC)_2_@TiO_2_	300 W Xenon (H_2_O)	2.64 μmol g TiO_2_ h^−1^ of CH_4_	Reuse
CdS/Co-ZIF-9	300 W Xe lamp (TEOA)	CO (50.4 μmol h^−1^), H_2_ (11.1 μmol h^−1^)	
g-C_3_N_4_+Co-ZIF-9	Xe lamp (H_2_O, CH_3_CN, TEOA)	20.8 μmol CO	Reuse
ZIF-8/Zn_2_GeO_4_	500 W Xe arc lamp	2.44 μmol g^−1^ CH_3_OH	XRD

**Table 5:** Summary of the photocatalytic H_2_ evolution based on non-noble-metal catalysts, with permission from [69].

Categories	Catalysts	Co-Catalysts	Merits	Demerits	H_2_ Production Rate (µmol.h^−1^g^−1^)
Metal oxide	TiO_2_	Doped N	Reduced microcrystalline rate and band gap	Low light absorption	1800
		Doped S	Reduced particle size, increased special surface area, better light capture	Hydrogen production activity reduced by sulfur ions	800
		Doped high content C	Improved lifetime of photogenerated carriers, widened light absorption range	Expensive raw materials	33.04
		Doped Fe and Ni	Widened light absorption range, improved electron–hole separation efficiency	An alcohol solvent is necessary for H_2_ production	361.64
		Doped Fe^3+^ and P^5+^	Enhanced charge separation	Narrow visible-light-response range	724
		Loaded NiO	Reduced band gap energy, more exposed area of TiO_2_	Glycerin is the main source of H_2_ production	1230
		Loaded NiS	Promoted fast carrier transport, more active sites	Limited reported examples	7486
		Loaded Cu	Inhibited electron–hole recombination	No H_2_ production on TiO_2_ without Cu	283
	ZnO	Doped S	Increased surface area, enhanced light capture	Complex preparation process	3640
		Doped Y and Al	Improved light absorption and stability, boosted electron transfer	Crystal size increases with the increase in Y	5710
		Loaded g-C_3_N_4_ NSs	Improved visible-light absorption and charge carrier separation, extended carrier life	Narrow visible-light-response range	322
		Coated MoS_2_	Favored charge transfer, inhibited electron–hole recombination	Only responds to ultraviolet light	768
		Loaded CdS QD_S_	Promoted photogenerated electron–hole separation and migration, inhibited electron–hole recombination, improved stability	High demand for morphology	22,120
Metal Sulfide	CdS	Loaded Ni_3_C NPs	Increased visible light absorption, promoted carrier separation and transfer, improved surface H_2_ production kinetics	Poor cycling stability	18,020
		Loaded Co_3_O_4_	Formation of p-n heterojunction; enhanced light capture, charge transfer, and separation capabilities	No H_2_ production compounded with P25, no stability test	3014
		Loaded TiO_2_	Formation of Z-scheme structure, promoted electron–hole separation	Limited cycle stability tests	1028
		Loaded MoS_2_/graphene	Synergetic effects, inhibited charge recombination	Complicated preparation process	1913
	ZnS	Doped Cu	Improved visible light absorption	Low oxidation potential	973.1
		Doped In and Cu	Improved visible light absorption, enhanced photogenerated charge transfer and carrier separation	Photocatalytic H_2_ production is not as effective	752.7
		Loaded ZnO/g-C_3_N_4_	Plentiful active sites, effective electron–hole separation	Narrow visible-light-response range	301.25
Metal Phosphides	NiP	Doped Mo	Electron filling can regulate the activity	Sacrificial agents are needed	15,300
		Doped Mn			16,700
		Doped Fe			17,000
		Doped Co			20,400
	Cu_3_P	Hybridized MoP	Construction of heterojunction, promoted carrier separation and transfer, expanded visible-light-response range	/	855
Metal Free	g-C_3_N_4_	Doped O	Reduced band gap, improved light capture	No stability test	348
		Doped P	Effective charge transfer	Reduced oxidation potential	427
		Doped N	Improved light harvesting, promoted photo-excited charge carrier separation and transfer, extended and delocalized π-conjugated system	Aromatic heterocycles contained	1280
		Doped B & F	Enhanced visible light absorption by B doping; promoted photoelectron generation, transfer, and separation by B–F co-doping and post-activation	Complicated preparation process, narrow visible-light response range	7020

**Table 6:** A comparative analysis of the common battery types.

Battery Type	Energy Density (Wh/kg)	Cycle Life (Cycles)	Self- Discharge	Applications	Advantages	Limitations	Ref.
Lead–Acid	30–50	200–300	5% per month	Automotive starting, lighting, and ignition (SLI); UPS	Low cost, mature technology, reliable	Low energy density, limited cycle life	[93]
Nickel– Cadmium (NiCd)	45–80	1000–2000	20% per month	Emergency lighting, power tools	Robust, fast charging capability	Memory effect, toxic materials	[94]
Lithium-Ion (LiCoO_2_)	150–250	1000–3500	5–10% per month	Mobile devices, electric vehicles	High energy density, low maintenance	Requires protection circuit, aging issues	[95]
Lithium Iron Phosphate (LiFePO_4_)	90–120	2000–5000	3–5% per month	Electric buses, solar storage	Very safe chemistry, long cycle life	Lower energy density than other Li-ion batteries	[96]
Sodium-Ion	80–150	2000–3000	10–15% per month	Grid storage, renewable integration	Low cost, abundant materials	Lower energy density, developing technology	[97]
Flow Batteries	20–40	12,000–14,000	Negligible	Grid storage, load leveling	Scalable capacity, long cycle life	Low energy density, system complexity	[98]

**Table 7:** The most famous metal oxides applications in energy storage technologies.

Metal Oxide	Key Properties	Applications	Ref.
Manganese Dioxide (MnO_2_)	High theoretical capacity, environmentally friendly	Alkaline batteries, supercapacitors, zinc-ion batteries	[102]
Lithium Cobalt Oxide (LiCoO_2_)	Stable layered structure, good lithium-ion conductivity	Lithium-ion batteries, portable electronics	[103]
Nickel Oxide (NiO)	High specific capacitance, excellent rate capability	Supercapacitors, hybrid supercapacitors, batteries	[105]
Vanadium Oxides (V_2_O_5_, VO_2_)	Versatile redox chemistry, structural flexibility	Flow batteries, cathode materials	[106]
Iron Oxides (Fe_2_O_3_, Fe_3_O_4_)	Abundant, low-cost, environmentally compatible	Sodium-ion batteries	[107]
Titanium Dioxide (TiO_2_)	Fast-charging performance, structural stability	Lithium-ion batteries, fast-charging batteries	[108]
Cobalt Oxide (Co_3_O_4_)	High theoretical capacity, stable cycling performance	Supercapacitors, batteries	[109]

**Table 9:** Overview of functionalized graphene for hydrogen storage: methods, media, and capacities.

Material	Functionalization Method	T (°C)	P (Pa)	Characterization	H_2_ Capacity (wt.%)	Ref.
Nb_2_O_5_/NG@MgH_2_	Heteroatom/Nanoparticle	170	5	Gravimetric	6.0	[23]
Ca-Graphene	Heteroatom doping	500	1	Gravimetric	4.98	[136]
Mg/rGO	Metal nanoparticle decoration	250	1.5	Volumetric	6.5	[137]
Ni/Graphene	Metal nanoparticle decoration	300	5.5	Volumetric	6.5	[138]
Ni-NP/Graphene	Metal nanoparticle decoration	25	0.5	Gravimetric	1.95	[139]
Graphene-TiO_2_	Metal nanoparticle decoration	25	0.5	Gravimetric	1.48	[141]
Cd_2_SiO_4_/Graphene	Metal nanoparticle decoration	25	0.1	Electrochemical	12.2	[137]
Co_2_SnO_4_ Graphene	Metal nanoparticle decoration	25	0.1	Electrochemical	10	[137]
Pd/N-Graphene	Heteroatom/Nanoparticle	25	4	Volumetric	4.40	[150]
Pd-rGO	Metal nanoparticle decoration	25	0.1	Electrochemical	0.11	[174]
Pd/B-rGO	Heteroatom/Nanoparticle	25	0.1	Electrochemical	0.33	[174]
Pd_3_P/P-rGO	Heteroatom/Nanoparticle	25	5.5	Volumetric	4.7	[175]
Pd/f-HEG	Metal nanoparticle decoration	25	2	Volumetric	1.76	[176]
Pt/SL-Graphene	Metal nanoparticle decoration	RT	0.1	Electrochemical	6.6	[177]
Li-Fe MOF/GO	MOF/Heteroatom	25	5	Volumetric	2.02	[178]
Pt/Graphene	Metal nanoparticle decoration	25	3	Volumetric	1.4	[179]
MgNi/N-Graphene	Heteroatom/Nanoparticle	100	–	Volumetric	5.4	[180]
Pd_3_Co/B-Graphene	Heteroatom/Nanoparticle	25	3	Volumetric	4.6	[181]
NG foam	Heteroatom doping	25	0.1	Electrochemical	7.1	[182]
SrCrO_4_/N-rGO	Heteroatom/Nanoparticle	25	0.1	Electrochemical	6.7	[183]
TiO_2_/N-rGO	Heteroatom/Nanoparticle	25	0.8	Gravimetric	0.91	[184]
N-Graphene	Heteroatom doping	25	0.1	Electrochemical	0.19	[185]
Co/Graphene	Metal nanoparticle decoration	25	0.1	Electrochemical	0.92	[186]

## Data Availability

Data are contained within the article.
